# A quantitative label-free analysis of the extracellular proteome of human supraspinatus tendon reveals damage to the pericellular and elastic fibre niches in torn and aged tissue

**DOI:** 10.1371/journal.pone.0177656

**Published:** 2017-05-18

**Authors:** Osnat Hakimi, Nicola Ternette, Richard Murphy, Benedikt M. Kessler, Andrew Carr

**Affiliations:** 1 Nuffield Department of Orthopaedics, Rheumatology and Musculoskeletal Sciences, University of Oxford, Oxford, United Kingdom; 2 The Jenner Institute, University of Oxford, Oxford, United Kingdom; 3 Target Discovery Institute (TDI) Mass Spectrometry Laboratory, Nuffield Department of Medicine, Oxford, United Kingdom; Mayo Clinic Minnesota, UNITED STATES

## Abstract

Tears of the human supraspinatus tendon are common and often cause painful and debilitating loss of function. Progressive failure of the tendon leading to structural abnormality and tearing is accompanied by numerous cellular and extra-cellular matrix (ECM) changes in the tendon tissue. This proteomics study aimed to compare torn and aged rotator cuff tissue to young and healthy tissue, and provide the first ECM inventory of human supraspinatus tendon generated using label-free quantitative LC-MS/MS. Employing two digestion protocols (trypsin and elastase), we analysed grain-sized tendon supraspinatus biopsies from older patients with torn tendons and from healthy, young controls. Our findings confirm measurable degradation of collagen fibrils and associated proteins in old and torn tendons, suggesting a significant loss of tissue organisation. A particularly marked reduction of cartilage oligomeric matrix protein (COMP) raises the possibility of using changes in levels of this glycoprotein as a marker of abnormal tissue, as previously suggested in horse models. Surprisingly, and despite using an elastase digestion for validation, elastin was not detected, suggesting that it is not highly abundant in human supraspinatus tendon as previously thought. Finally, we identified marked changes to the elastic fibre, fibrillin-rich niche and the pericellular matrix. Further investigation of these regions may yield other potential biomarkers and help to explain detrimental cellular processes associated with tendon ageing and tendinopathy.

## Introduction

The supraspinatus tendon is part of the four rotator cuff tendons acting to stabilize the shoulder. The tendon proper is a viscoelastic and highly hierarchical tissue with collagen as the primary component of the extracellular matrix (ECM), organised in fibrils and fascicles surrounded by proteoglycan rich material [1]. Collagen is thought to account for 65–80% and the fibrous protein elastin for approximately 1–2% of the dry mass of tendon in general [2], with the latter described to occur in elastic fibres approximately 0.3–2.0*μ*m in diameter, broadly distributed throughout the tendon, localized longitudinally along cell arrays and between collagen fascicles [3]. The supraspinatus tendon is known to be poorly vascularised [4], with cell arrays embedded in a Collagen VI rich pericellular matrix [5]. Tears of this tendon are very common and are often painful and debilitating. The management of rotator cuff disease represent an increasing social and economic burden [6, 7]. The progression of rotator cuff disease is accompanied by numerous pathological changes [8], and various studies have sought to understand these by measuring differences between healthy and torn supraspinatus tendons. Past investigations have revealed changes in gene expression [9], ultrastructure [10] and the presence of specific biochemical markers [11]. Moreover, studies quantifying pre-specified mRNA molecules and proteins have shown collagen I and III, MMP-1, -3, -9, and -13, TIMP-1, and VEGF to be differentially abundant in human tendinopathy, but the link to rotator cuff tears remains uncertain [12]. To date, no studies using non-targeted, label-free protein quantitation to evaluate differential abundance of proteins between torn and healthy human rotator cuff supraspinatus tissue have been reported. The potential advantages of a non-targeted proteomics approach is the generation of a more complete inventory of proteins present in the tissue, the ability to quantitatively compare their abundance in the sample pool, and the possibility to identify proteins that display a significant fold-change and a biological likelihood of being candidate biomarkers. In here, grain-size biopsies of the supraspinatus tendon were homogenised and entirely digested by trypsin, with a parallel elastase digestion used as a validation and to complement and improve the proteome. This study reports the first ECM inventory of human supraspinatus tendon in health and disease, generated employing a label-free LC-MS/MS analysis.

## Materials and methods

### Donors and biopsies

The 27 patients who donated tissue for the study were treated either for a shoulder stabilisation in which there is no tendon abnormality or a tendon tear (see Table 1). Torn samples were obtained from patients with established tendon disease, presenting pain, full-thickness rotator cuff tears and scheduled to undergo surgery. Donated tissue were rice-grain sized end of biopsies, of around 2x1mm, collected using a minimally invasive ultrasound guided technique as previously described [13]. Tissue biopsies acquired using the same procedure were previously characterised by histological examination in several studies, demonstrating that diseased supraspinatus tissue was accurately collected [11, 14, 15]. After removal, biopsies were placed in 0.5ml Eppendorf tubes, snap-frozen in liquid nitrogen and stored at −80°C. Approval for the study was obtained from the Oxfordshire Research Ethics Committee (REC-B). All methods (tissue collection and storage) were performed in accordance with the relevant guidelines and regulations of the Oxford Musculoskeletal BioBank (OMB, ethics ref: 09/H606/11). All patients gave informed consent prior to tissue donation.

**Table 1:** Demographics of tendon donors.

Group	No of patients	Sex	Mean Age(SD)
Male Control, normal tendon	9	Male	24.44 (2.62)
Female torn tendon	9	Female	57.44 (8.7)
Male torn tendon	9	Male	58.3 (8.4)

### Protein extraction and digestion from tendon tissue

Rice-grain sized tendon tissue biopsies, approximately 1x2mm in size, were rinsed with cold phosphate buffered saline to remove residual blood proteins, transferred to screw-cap vials (M-tubes, Miltenyi Biotec) and suspended in 1.5 ml of strong chaotropic solution for 2 hours at room ambience (4M GuHCl, 10mM EDTA, 10mM DTT, 44mM sodium acetate). To homogenise the tissue biopsies, samples were vortexed and processed using the gentleMacs dissociator (Miltenyi Biotec), an automated tissue homogeniser. Thereafter, samples were centrifuged at a low speed for 5 minutes to collect dissociated tissue from the rotor/stator cup of the tube. The homogenisation procedure was repeated twice. Tubes were briefly vortexed and 0.5ml of each sample was transferred to a low-binding microcentrifuge tube and centrifuged for 10min at 14,000xg and 4°C. Protein material was then precipitated using Methanol/Chloroform [16]. Resulting protein pellets were re-suspended in 100*μ*l 6 M Urea in 100 mM Tris, pH 7.8. Following reduction with 10 mM DTT, cysteine residues were alkylated using a final concentration of 40 mM iodoacetamide for 30 min at room temperature. Samples were diluted with water to a final urea concentration of 0.67 M, and either trypsin (0.66ug/sample) or elastase (1ug/sample) were added. Digestion was carried out at 37°C overnight, after which samples were observed to be completely solubilised. Following acidification with formic acid (0.1% final concentration), obtained peptides were purified on a C18 reverse phase column (SepPak, Waters), dried and re-suspended in 20*μ*l 2% acetonitrile, 0.1% formic acid in water.

### Label-free liquid-chromatography mass spectrometric analysis

Each biological sample was analysed in duplicates. In order to make sure equivalent amounts of protein were analysed from each sample, total ion counts (TIC) was calculated using scout runs, and values were used to adjust the injection volume for the analytical sample runs. Equal amounts of peptide material were analysed by UPLC (nano Acquity, Waters) online coupled to an LTQ Orbitrap Elite (Thermo Scientific) as described previously [17]. Peptides were eluted by applying a 60 min linear gradient from 3% buffer A (0.1% formic acid in water) to 40% buffer B (0.1% formic acid in acetonitrile) on a 25 cm BEH130 C18 column, 1.7-mm particle size (Waters). Collision-induced dissociation (CID) was induced on the twenty most abundant ions per full MS scan.

### Database searches and quantitative proteome analysis

All mass spectra of label-free samples were extracted by Progenesis LC-MS, and then analysed by Mascot (Matrix Science), which was set up to search against all human Swiss Prot database entries with the following parameters: Trypsin or no enzyme search for trypsin and elastase samples, respectively; fixed modification: carbamidomethylation (cysteine), variable modifications: Oxidation (proline, lysine) and allysine (lysine). Normalization between samples was achieved using the total ion intensities (TIC) of each individual sample. Criteria to ensure accurate identification of tendon proteins included a minimum of 2 peptides per protein and a confidence score cut-off of 20. One-way ANOVA was used to calculate the p-value based on the normalized abundance, and a Tukey post-test was performed, providing an adjusted P value to account for multiple comparisons. An adjusted ANOVA score of at least 0.05 between experimental groups was used to identify proteins significantly modulated in torn tendon. Proteins were grouped based on their protein class using the PANTHER database of protein families (version 10) and the Matrisome project, and protein interactions were identified using STRING version 10.0.

### Independent verification of COMP by western blot

In order to confirm the alteration in COMP expression, as identified by label-free mass spectrometry, immunoblot analysis was employed. Electrophoretic separation of proteins from the extracts pre enzymatic digestion was performed using gradient polyacrylamide gels (Criterion XT Pre-cast 4–12% Bis-Tris gel, 26 well) and a prestained molecular weight marker (Precision Plus Protein Standard All Blue, Biorad) followed by wet transfer at 100 V for 70 min at 4°/C to Immobilon-P PVDF membrane (Millipore, Billerica, USA) in a Transblot Cell from Bio-Rad Laboratories (Hemel-Hempstead, Hertfordshire, UK). Membranes were blocked for 1 hour at room temperature with Rotiblock (Carl-Roth, Karlsruhe, Germany) prior to incubation with the primary antibody (anti-COMP, ab74524, abcam UK) at a dilution of 1:1000 in Rotiblock, overnight at 4°C with gentle agitation. Membranes were subsequently washed twice with PBS containing 0.05% Tween 10 min each time, followed by incubation for 1 h with polyclonal horseradish peroxidase-conjugated goat-anti-mouse secondary antibody (P0447, Dako, Ely, UK) at a dilution of 1:10,000 in Rotiblock. Visualisation of antibody-labelled protein bands on washed membranes was achieved using Amersham ECL Prime Western Blotting Detection Reagent (GE Healthcare, UK) according to the manufacturer’s guidelines.

## Results

**Fig 1:**
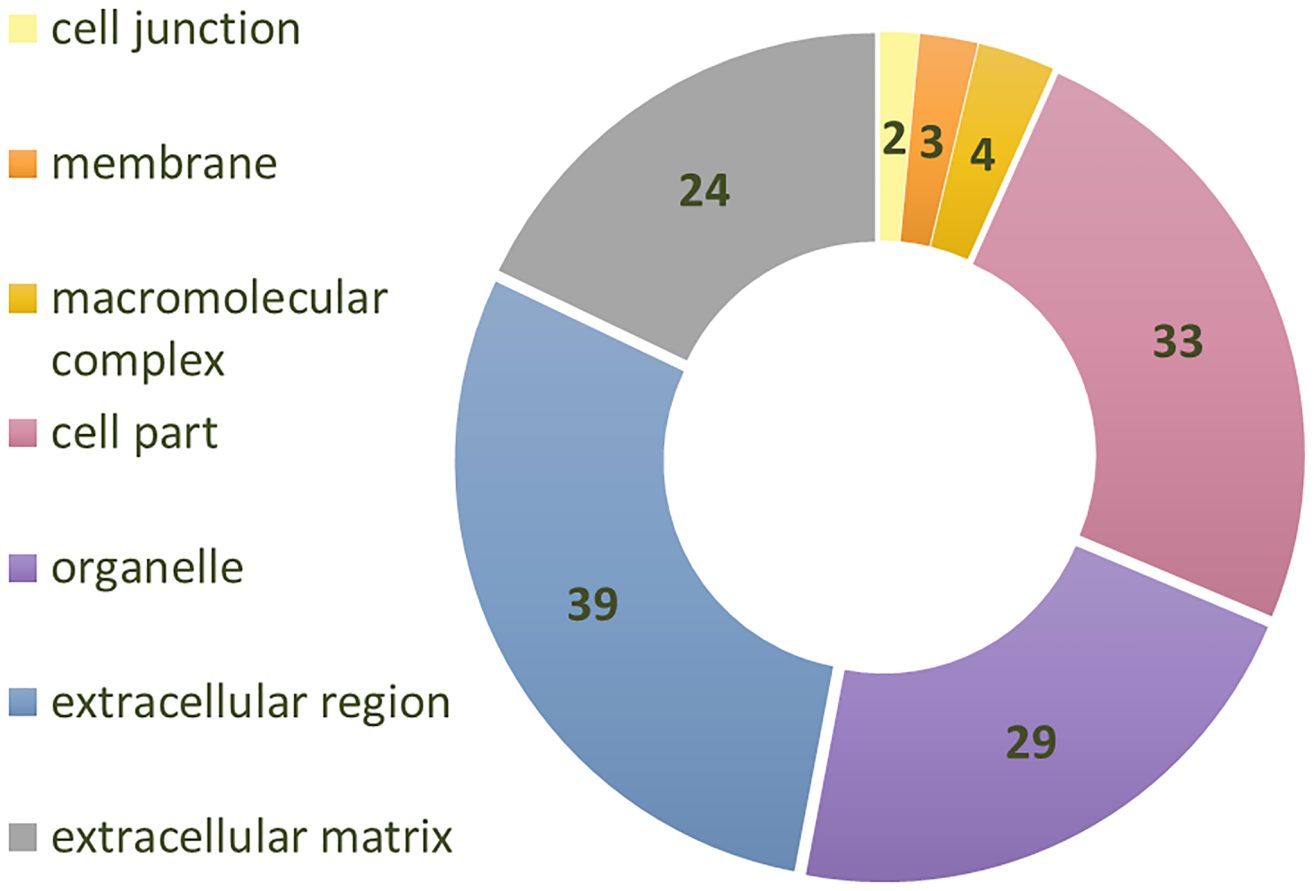
The number of proteins in each cellular compartment of the proteome extracted from supraspinatus tendon samples using tryptic digestion. 126 identified proteins (Progenesis, at least 2 identified peptides) were categorized using PANTHER classification system.

Samples analysed in this study consisted of tendon supraspinatus biopsies from 27 donors belonging to three groups: aged and torn male donors (n = 9), aged and torn female donors (n = 9), and healthy and young male donors (n = 9), the later used as control. Significant differences in key tendon structural components were expected between healthy/young and aged/torn rotator cuff tissue. A total of 145 proteins were identified, but only 126 of these, with at least two identified peptides, were considered for the analysis. Panther classification system was used to sort proteins by compartments (see Fig 1). Out of the 126 proteins identified, 63 were extracellular matrix proteins, or proteins predominantly found in the extracellular region. This number is similar to a previous study of human patellar tendon, which has identified 166 proteins, but only 68 proteins with at least 2 peptides [18].

Extracellular proteins which were identified in trypsin-digested samples are summarised in Table 2, and the accession number and number of peptides identified for each protein are given, as well as an indication if the protein was also identified in the elastase-digested samples. To give a simplified overview of the relative abundance of identified proteins (RA, see Table 2), the mean percentage RA of each protein compared to COL1A1 was calculated for the control group. It should be noted that these ratios represent the relative abundance of these proteins in the trypsin-digested fraction of the normal tissue samples, and not an absolute value for supraspinatus tendon. Moreover, values were normalised to just one subunit of Collagen I, rather than total collagen content. As can be seen in Table 2, five fibrillar collagens (I, II, III, V and XI) and three non-fibrillar collagens (VI, XV, XIV) were identified. The number of peptides identified for Collagen I and II was significantly higher than the average of around 90 previously reported (Sejersen et al. 2015). Among the non-fibrillar collagens, collagen VI was the most abundant, and also had a much higher number of peptide hits per subunit. In addition to collagens, 30 matricellular proteins were identified in the trypsin digested samples. These included three elastic fibre component proteins (fibrillin 1, fibrillin 2 and microfibillar associated protein 5), but not elastin, which was not detected in neither trypsin nor elastase digested samples. Seven members of the small leucine-rich proteoglycans (SLRPs) family were also identified and three of the thrombospondin family. The three most abundant matricellular proteins were cartilage oligomeric matrix protein (COMP), decorin, and fibrillin-1.

**Table 2:** Identified collagens and matricellular proteins, showing accession number, name, peptide count and relative abundance (RA) as mean percentage of COL1A1 in young, healthy samples. ES stands for elastase samples, and a (+) showing if proteins were identified in elastase digested samples. NQ is non-quantified, and (*) is also known as microfibril-associated glycoprotein-2 (MAGP-2).

Classification	Family/Sub-group	Accession umber	Name	Protein symbol	Peptide count (unique)	Percentage of Col1A1 (RA)	ES
Collagens	Fibrillar collagens	P02452	Collagen alpha-1(I) COL1A1	230 (216)	100	+	
		P08123	Collagen alpha-2(I)	COL1A2	109 (103)	53.47	+
		P02458	Collagen alpha-1(II)	COL2A1	17 (8)	0.05	-
		P02461	Collagen alpha-1(III)	COL3A1	138 (135)	40.35	+
		P20908	Collagen alpha-1(V)	COL5A1	25 (20)	0.87	+
		P05997	Collagen alpha-2(V)	COL5A2	29 (26)	0.97	+
		P12107	Collagen alpha-1(XI)	COL11A1	6 (2)	0.08	-
	Non-fibrillar collagens	P12109	Collagen 1(VI)	COL6A1	33 (29)	1.56	+
		P12110	Collagen 2(VI)	COL6A2	28 (24)	0.85	+
		P12111	Collagen 3(VI)	COL6A3	80 (75)	1.11	+
		Q05707	Collagen alpha-1(XIV)	COL14A1	16 (12)	0.005	-
		P39059	Collagen alpha-1(XV)	COL15A1	2 (2)	0.01	+
Matricellular proteins	Elastic fibre component	P35555	Fibrillin-1	FBN1	46 (43)	1.37	+
		P35556	Fibrillin-2	FBN2	3 (0)	NQ	-
		Q13361	Microfibrillar-associated protein 5*	MFAP5	3 (3)	0.07	-
		Q14767	Latent-transforming growth factor beta-binding protein 2	LTBP2	4 (4)	0.03	+
	Small leucine-rich proteoglycans (SLRPs)	P07585	Decorin	DCN	30 (28)	4.13	+
		P20774	Mimecan	OGN	8 (7)	0.02	-
		P51884	Lumican	LUM	17 (16)	0.5	+
		Q06828	Fibromodulin	FMOD	10 (10)	0.18	+
		P21810	Biglycan	BGN	34 (31)	0.83	+
		Q9BXN1	Asporin	ASPN	13 (13)	0.2	-
		P51888	Prolargin	PRELP	20 (18)	0.55	-
	Lectican	P13611	Versican core protein	VCAN	21 (21)	0.26	+
		P16112	Aggrecan core protein	ACAN	11 (10)	0.008	-
	Tenascin	P22105	Tenascin-X	TNXB	72 (68)	1.09	+
		P24821	Tenascin	TNC	10 (9)	0.01	+
	Fibulin	P23142	Fibulin-1	FBLN1	3 (3)	0.02	-
	Thrombo-spondins	P49747	Cartilage oligomeric matrix protein	COMP	43 (41)	6.57	+
		P35443	Thrombospondin-4	THBS4	10 (8)	0.08	+
	CILP	O75339	Cartilage intermediate layer protein 1	CILP	28 (23)	0.94	+
		Q8IUL8	Cartilage intermediate layer protein 2	CILP2	31 (26)	1.25	+
	Fibronectin type III domains	Q7Z7G0	Target of Nesh-SH3	ABI3BP	9 (8)	0.06	-
		P02751	Fibronectin	FN1	12 (12)	0.07	+
	Other	Q15063	Periostin	POSTN	10 (10)	0.006	+
		P02765	Alpha-2-HS-glycoprotein	AHSG	2 (2)	0.008	-
		Q99972	Myocilin	MYOC	13 (13)	0.34	-
		Q92954	Proteoglycan 4 (Lubricin)	PRG4	5 (4)	0.01	+
		P04004	Vitronectin	VTN	2 (2)	0.02	+
		P05452	Tetranectin	CLEC3B	2 (2)	0.002	+
		Q07507	Dermatopontin	DPT	3 (3)	0.02	-

**Fig 2:**
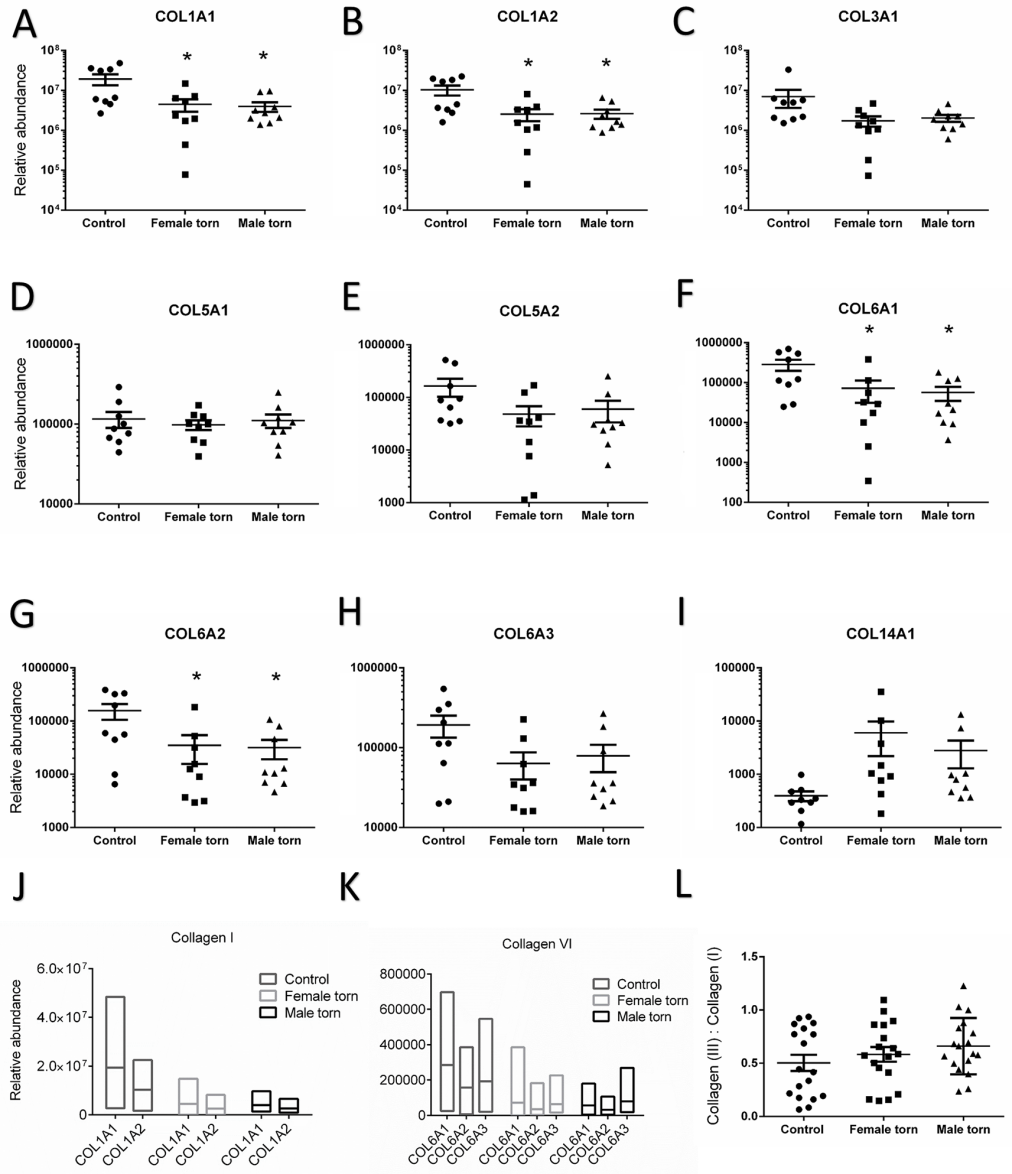
The relative abundance of collagens and their subunits in supraspinatus tendon from control and pathologic female and male donors. Dot plots show biological replicates, means and standard error of the mean. (A) COL1A1, (B) COL1A2, (C) COL3A1, (D), COL5A1, (E) COL5A2, (F) COL6A1, (G) COL6A2, (H) COL6A3 and (I) COL14A1. (J) Floating bars showing minimum and maximum relative abundance of the subunits of collagen I with a line at the mean and (K) the relative abundance of the subunits of collagen VI. (L) The ratio of collagen III to I, average of ratios in individual runs within each group (duplicates per sample, n = 18 per group). *P<0.05, **P<0.01 significantly different from control.

**Fig 3:**
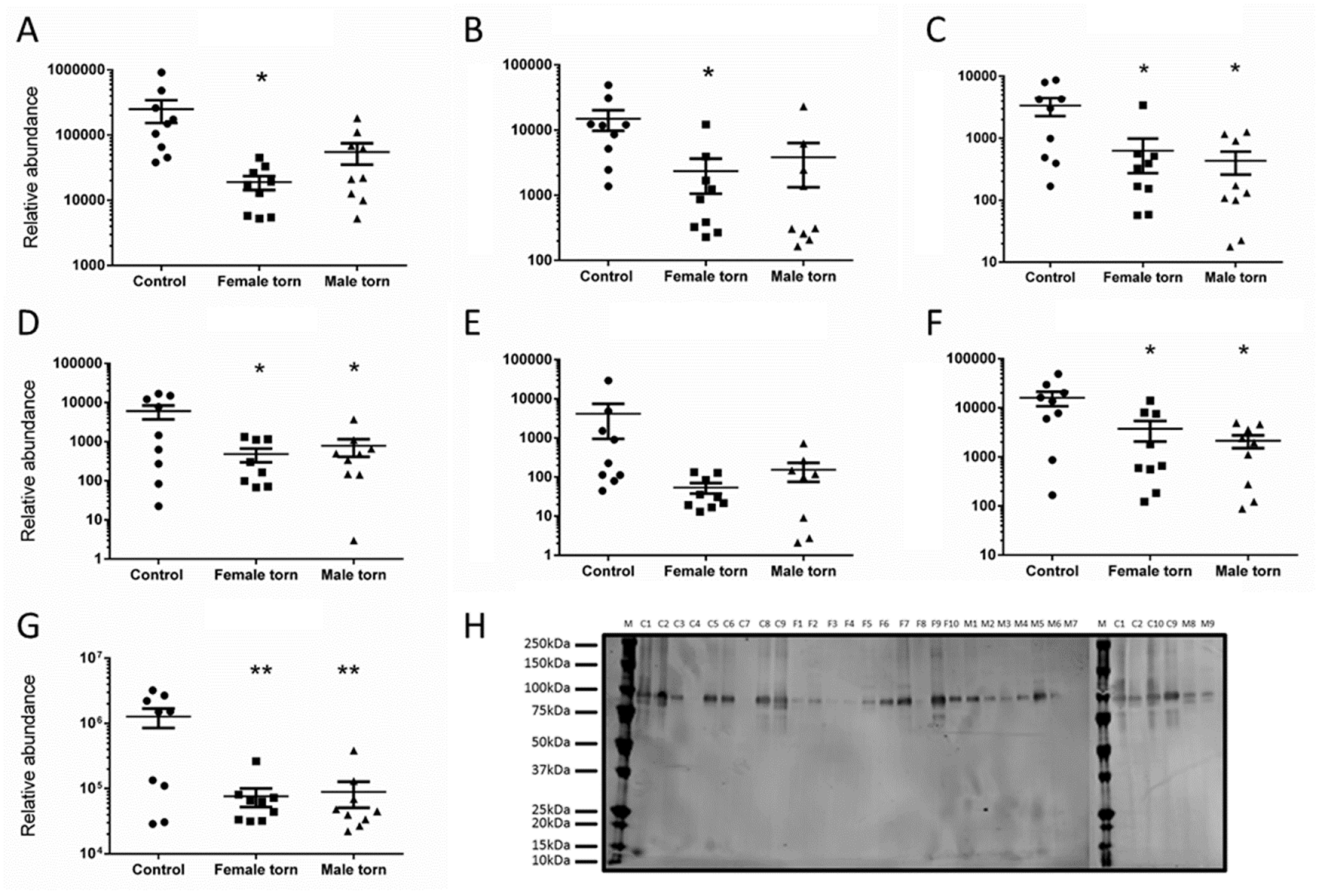
The relative abundance of differentially expressed elastic fibre components (A-D) and members of the thrombospondin family associated proteins (E-G). Dot plots show biological replicates, means and standard error of the mean. (A) fibrillin-1 (FBN1), (B) microfibrillar associated protein 5 (MFAP5), (C) Fibulin-1 (FBLN1), (D) Latent-transforming growth factor beta-binding protein 2 (LTBP2), (E) Thrombospondin-1, (F) Thrombospondin 4, and (G) cartilage oligomeric protein (COMP). (H) Western blot of COMP (around 100kDa), confirming higher prevalence in control compared to female and male torn and aged tendon samples. *P<0.05, **P<0.01 significantly different from control.

Table 3 shows all collagens and matricellular proteins which varied significantly between torn and control tendon samples. There were no statistically significant differences of protein abundances between the torn samples from male and female groups. Collagens I and VI, but not III, were differentially abundant between control and torn samples. The ratio between collagen I and III, often used as a marker of tendon tissue repair, was not statistically different between healthy and torn samples, but the ratios between two of the three subunits of collagen VI (COL6A2 and COL6A3) were significantly different in healthy tendon (Table 4). Ten matricellular proteins were statistically lower in torn samples (see Table 3), and this reduction was observed in torn samples from both male and female donors. Proteins of interest are also presented individually below in Figs 2 and 3, in order to demonstrate the spread of the data and ratios between the subunits and related proteins. In terms of variability, inter-patient variability can be seen by the spread of data points in Figs 2 and 3. However, because a single and very small tissue sample was processed from each donor, any intra-patient variability is due to technical rather than biological variation. All proteins presented in Table 3 were significantly lower in torn samples compared to control. Overall, approximately 30% of the proteins detected were more abundant in torn samples compared to control, but only four of them significantly (S1 Table). None of the proteins upregulated in the torn samples was an extracellular compartment protein.

**Table 3:** The ANOVA P value and mean fold change of differentially expressed extracellular proteins in the torn tissue of female and male patients compared to control in the trypsin digested samples. (*P <0.05, **P <0.01, significantly different from control in post-test).

Protein	ANOVA P Value	Mean fold change F torn/Control	Mean fold change M torn/Control
COL1A1	0.0218	0.23*	0.20*
COL1A2	0.0164	0.24*	0.25*
COL6A1	0.0156	0.25*	0.20*
COL6A2	0.0300	0.22*	0.20*
FBN1	0.0175	0.08*	0.22
MFAP5	0.0301	0.16*	0.26
COMP	0.0021	0.06**	0.07**
THBS4	0.0096	0.23*	0.13*
CILP	0.0260	0.16*	0.30
CILP2	0.0095	0.08*	0.06*
LTBP2	0.0136	0.08*	0.13*
FBLN1	0.0074	0.19*	0.13*
TNXB	0.0021	0.14**	0.35*
MYOC	0.0157	0.11*	0.22*

**Table 4:** The ratios of COL3A1 to COL1A1, and of the subunits of Collagen I and Collagen VI in the three groups, Mean(SEM), *P<0.05 **P<0.01 significantly different from control.

	Control	Female torn	Male torn
COL3A1 : COL1A1	0.502 (0.076)	0.583 (0.068)	0.66 (0.062)
COL1A1 : COL1A2	1.745 (0.065)	1.699 (0.044)	1.511 (0.079)*
COL6A1:COL6A2	2.243 (0.29)	1.786 (0.277)	1.722 (0.184)
COL6A3:COL6A2	1.675 (0.231)	3.309 (0.529)**	2.944 (0.215)*

Of the ten matricellular proteins significantly lower in torn samples (listed in Table 3), four are strongly linked to elastic fibres (see Fig 3A–3D). Of these four, three are elastic fibre components (FBN1, MFAP5, and LTBP2), and one is known to be associated with elastic fibres (FBLN1) [19, 20]. A significant lower abundance of LTBP2 in torn and aged samples was also identified in the elastase-digested samples (Table 5). FBLN2, another elastic fibre component previously reported in canine tendons [21] was identified but was not quantified because of a low peptide count. Of the three members of the thrombospondin family identified here, two (COMP and THBS4) were statistically lower in torn tendons compared to control (Fig 3F and 3G). Among the matricellular proteins, cartilage oligomeric matrix protein (COMP) was reduced with the highest probability (ANOVA P = 0.0021, adjusted for multiple comparisons: 0.0052 and 0.0057 for female and male, respectively). This reduced abundance was further validated using western blot (Fig 3H) and in the elastase-digested samples (Table 5).

**Table 5:** The mean relative abundance of differentially expressed extracellular proteins in the elastase digested samples, mean (SEM), (*P<0.05 **P<0.01 significantly different from control).

Protein	Peptide count (unique)	ANOVA P Value	Fold change F torn/Control	Fold change M torn/Control
COMP	48 (36)	0.0236	0.09*	0.11*
CILP	29 (26)	0.0258	0.15*	0.16*
CILP2	32 (29)	0.0209	0.11*	0.04*
LTBP2	6 (6)	0.0178	0.25*	0.34*
TNXB	26 (25)	0.0022	0.25**	0.23**

### Elastase digestion

**Fig 4:**
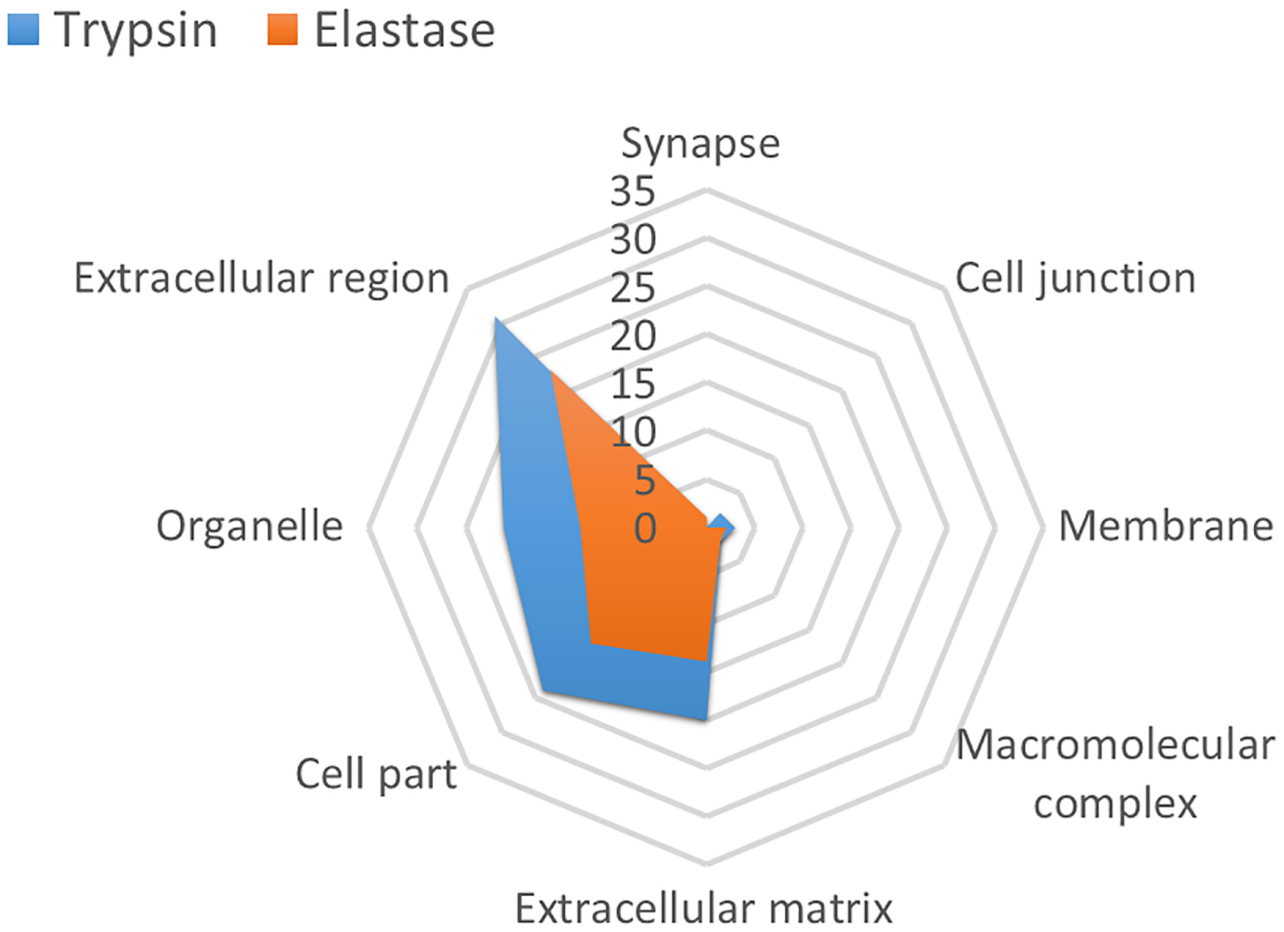
The number of hits in each cellular compartment in samples digested by either trypsin (blue) or elastase (orange). Identified proteins (Progenesis, 2 or more peptides) were categorized using PANTHER classification system. Although tryspin digestion yielded more hits, there was a similar distribution of proteins in the compartments.

Since many ECM proteins contain sequences lacking lysine and arginine residues and are therefore refractory to trypsin mediated proteolysis, we utilized elastase to complement and expand the tendon proteome. In total, 79 proteins were identified in the elastase-digested samples (with at least two peptide hits), much lower than the 126 proteins identified in the tryptic digestion batch. 29% of the proteins identified were unique (see S2 Table), whereas 71% overlapped with the trypsin-digested samples. The extracellular compartment predictably dominated the elastase-digested proteome, and overall the distribution of identified proteins in the different cellular compartment was very similar to trypsin-digested proteome (Fig 4). One major discrepancy between the proteomes of the trypsin and elastase digestions was the inverted abundance ratio of the collagen I subunits (COL1A1 and COL1A2), as well as a much lower number of unique peptides identified for COL1A1 (70 unique peptides in elastase digested samples and 216 in trypsin digested samples). The ratios between the subunits of collagen VI were also fairly different, although there was an increase in the ratio of COL6A3:COL6A2 in torn samples (see S3 Table), similar to the trend observed in trypsin digested samples. The abundances of none of the collagen superfamily were significantly different between the torn and control groups. Five matricellular proteins (COMP, CILP1, CILP2, LTBP2 and TNXB, see Table 5) had a significantly lower abundance in torn samples, similarly to the trypsin-digested samples. Interestingly, and despite using elastase for its specific ability to digest elastin, no elastin peptides were identified in any of the 27 samples analysed. To ensure that the absence of elastin was not due to an error in the protocol, rat aorta was digested as a control using the same protocol, and elastin was detected with a protein score of 2093 and 57% coverage (see S1 Appendix). Taken together, and unless there were some solubility issues specific to elastin in this tissue, these findings suggest that elastin is not as highly abundant in human supraspinatus tendon as previously suggested for tendon in general [2]. Other proteins highly relevant to tendon development and disease were only identified in the elastase digested samples, including Cadherin 11 (CDH11), an important tendon development cell-surface marker, and hydrocephalus-inducing protein homolog (HYDIN), which was statistically lower in torn samples, and has been previously linked to cilia dysfunction related diseases. A full list of proteins uniquely identified in the elastase-digested samples is in S2 Table.

## Discussion

This study is the first inventory of the proteome of human supraspinatus tendon ECM from young, healthy patients and an older cohort of patients with rotator cuff tears. A major advantage of this study is the use of elastase digestion in parallel to trypsin, providing an interesting comparison and a non-targeted validation. The findings presented here provide a confirmation of significant changes to at least five proteins already studied in ageing and injured tendon tissue, as shown in Table 6.

**Table 6:** Previously published studies confirming the disruption of COL1, COL6, FBN1, COMP and LTBP2 in torn and/or aged tendons.

Protein	Findings from previous studies
COL1	Highly organised collagen matrix is replaced by aberrant collage in human RC [22] Degenerate human supraspinatus and subscapularis tendons had a reduced collagen content [23]
COL6	Type VI collagen transcription was upregulated in human rotator cuff tears [5]
FBN1	Fibrillin 1 transcription was upregulated in human rotator cuff tears [5]
COMP	Equine tendon COMP is reduced with age [26] Specific cleavage patterns of COMP in injured horse tendon [25]
LTBP2	TGF-beta superfamily members were dysregulated in diseased compared to healthy tendons [11]

A clear downregulation of collagens has been previously described in torn supraspinatus tendon [22–24] and in particular a significant reduction in the abundance of both of the subunits of collagen I. Moreover, a significantly lower abundance of cartilage oligomeric matrix protein (COMP) was measured in torn samples. COMP was the most abundant matricellular protein detected in this study, and its reduced abundance was also observed in the elastase digested samples and by western blot. A similar reduction of COMP in injured tendon has been previously reported in the horse superficial digital flexor tendon [25]. It is also known that COMP levels fall with age [26]. As COMP is known to co-localise with collagen I [27], this quantitative analysis supports the assumption that the torn tendons in older patients have suffered a significant degradation of collagen I fibrils. Surprisingly, no significant change of collagen III was measured. Indeed, there was a non-significant increase in the ratio of collagen I and collagen III, in line with previous findings from total collagen measurements in cadaver supraspinatus [23], but overall the abundance of collagen III was slightly lower (although not significantly) in the older, torn samples. This is an interesting issue, and a recent systematic review of the literature has made a claim that the expression of collagen III is increased in tendinopathy [12]. Although this claim is well supported by transcriptome data [28–31], protein evaluation directly from torn supraspinatus has resulted in mixed results, with one study showing an increase in the ratio of collagen III to collagen I [23] and another a reduction in both collagens [24]. This calls for some consideration of the possibly significant differences between transcriptome and proteome data, with processes such as degradation or the presence of complex post-transcriptional regulation pathways affecting the actual composition of the tissue. Additionally, a recent detailed study of collagen distribution in the supraspinatus tendon has shown that the content of collagen III is homogeneously distributed, and not specifically localised in regions where tears are known to initiate [32]. Thus, it is possible that the previously observed increase in the relative abundance of collagen III is the result of a more pronounced decrease in collagen I, as we observed in our quantitative analysis, although further work is required to ascertain this point.

**Fig 5:**
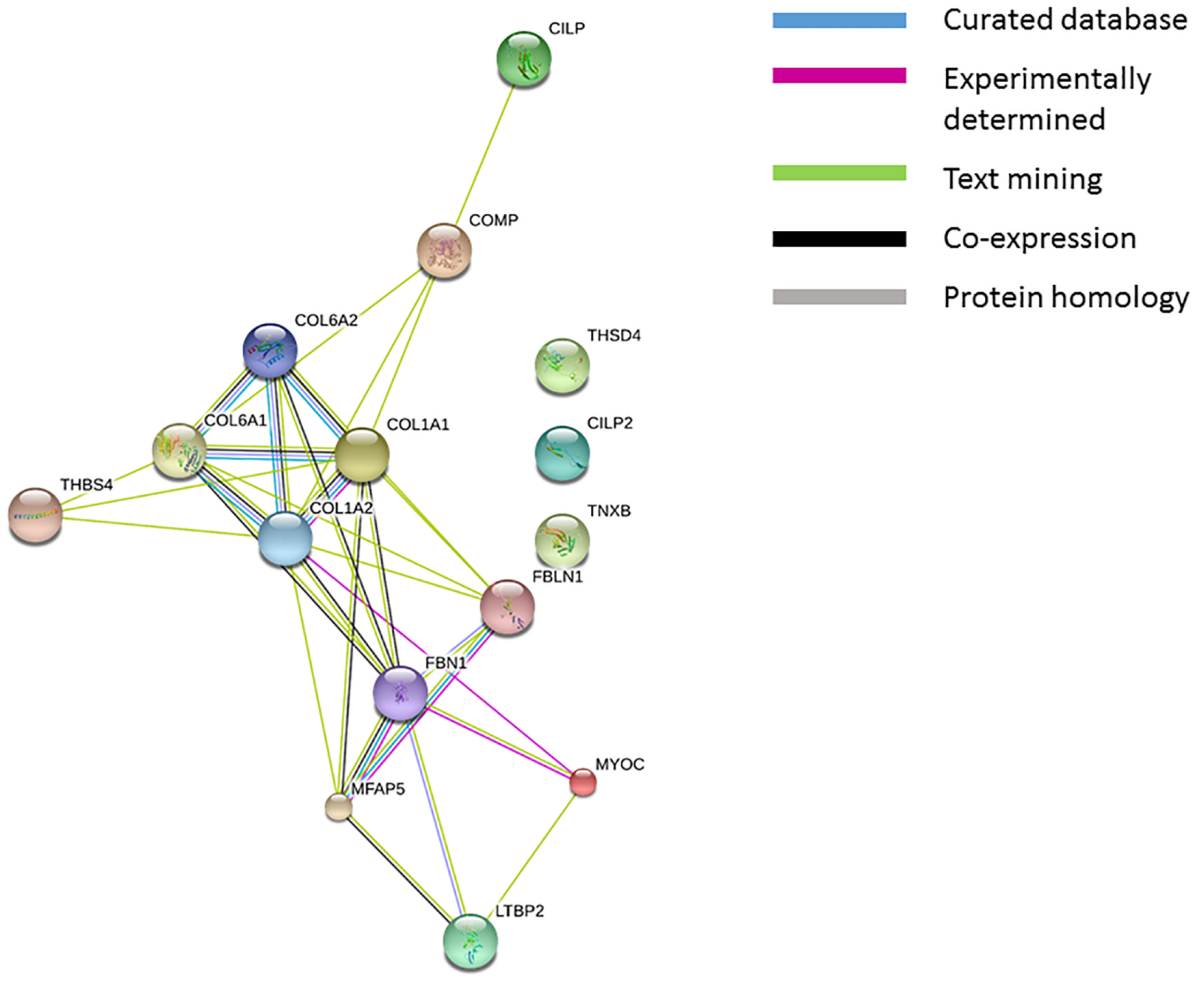
The interactions of all proteins which were significantly reduced in torn samples compared to control, created using the STRING database. The different line colours represent the different types of associations between the differentially modulated proteins, which include known interactions such as those identified in the curated database (light blue), and experimentally determined (purple). Other interactions identified were via text mining (green), co-expression (black) and protein homology (grey). Proteins are: COL1A1, Collagen 1(I); COL1A2, Collagen 2(I); COL6A1, Collagen 1(VI); COL6A2, Collagen 2(VI); FBLN1, Fibrillin-1; MFAP5, microfibrillar associated protein 5; COMP, Cartilage oligomeric protein; THSD4, Thrombospondin-4; CILP1, Cartilage intermediate layer protein 1; CILP2, Cartilage intermediate layer protein 2; TGFB1, Latent-transforming growth factor beta-binding protein 2; FBN1, Fibulin-1; TNXB, Tenascin-X; MYOC, Myocilin.

At least four proteins associated with elastic fibres were significantly less abundant in the torn and aged samples: fibrillin-1 (FBN1), microfibrillar associated protein 5 (MFAP5), Fibulin-1 (FBLN1), and Latent-transforming growth factor beta-binding protein 2 (LTBP2). No elastin has been detected in any samples, neither in the trypsin nor the elastase digested tissue, and its absence in the latter requires further investigation and explanation. The large glycoprotein FBN1 was the third most abundant matricellular protein identified in this study, consistent with its role as the main structural component of beaded microfibrils. It is known to interact directly with MAFP5 [33, 34], as well as LTBP2, with the latter targeting and sequestering TGF-*β* to the fibrillin-microfibril niche, as well as binding fibulins [35]. FBLN1 has been shown to co-localise with elastic fibres, but there is little information about its exact role [20, 36] The existence of fibillin-rich microfibrils in tendon has previously been described in fetal calf achilles [33], canine flexor digitorum profundus [37], bovine flexor tendon [3], and in human supraspinatus tendons, where they have been shown to be regularly distributed and in the vicinity of tendon cell arrays [5], with all these studies showing elastin to co-localise with the fibrillin microfibers. It is curious, therefore, in light of the high abundance of fibrillin-1 detected here and the use of elastase digestion as validation, that we did not detect any elastin peptides. There is a possibility that elastin in supraspinatus tendon is cross-linked or modified in a manner which renders it difficult to extract or insoluble. However, one theory worth exploring is that fibrillin-microfibrils in supraspinatus tendon are elastin-free, similarly to the ones described in the periodontal ligament [38]. An in situ hybridization study of the localised expression of ECM components in healing supraspinatus tendon in the rat has found elastin to be present only in blood vessels [39], and a recent study in the horse superficial digital flexor tendon has shown elastin was predominantly localised to the interfascicular matrix [40], whilst it is well established that fibrillin-rich microfibrils are regularly interspersed throughout the fascicular matrix. Regardless of the absence of elastin, the observation that four different elastic fibre components were significantly less abundant in torn tendons, suggests a systematic damage to the fibrillin-rich microfibril niche. This is supported by a previous study, which observed a disturbance to fibrillin-rich elastic fibres in large and massive supraspinatus tears using confocal microscopy [5]. The impairment of this micro-environment, which serves as a repository for growth factors, and in particular TGF-*β* [35], may also help to explain the disruption of the TGF-*β* axis in tendinopathy [11]. In light of the important role of the fibrillin-rich microfiber in tendon, not just as a structural component of the ECM, but as a network of cell and matrix interactions with considerable effects on cell function [41], it will be of interest to resolve the question of its exact composition in the supraspinatus tendon. The clear and direct interaction of this niche with collagen fibrils and the pericellular region is demonstrated in Fig 5.

In terms of the pericellular region, at least two proteins localised at this niche were altered in aged, torn tendon: collagen VI and thrombspondin-4 (THBS4) [42, 43]. Collagen VI is a non-fibrillar collagen known to form highly branched, beaded filamentous networks which encircles collagen fibrils and is particularly abundant in the pericellular region around cell arrays in several tissues, including muscle and tendon [21, 44]. It is composed of three different peptide chains COL6A1 and COL6A2 and COL6A3, the latter much larger and thought to be responsible for the intracellular assembly of the subunits [45]. In this study a statistically significant reduction in the abundance of subunits COL6A1 and COL6A2, and a significant change to the ratios of the subunits between the young and older, torn tendons was found. This differential expression of the subunits has been shown before in vitro, with COL6A1 and COL6A2 but not COL6A3 upregulated in the presence of three- dimensional collagen I gel [46]. Interestingly, whilst the reduction in collagen VI in torn, aged tendon observed here is in accordance with a recent proteome study in a horse model [47], it is contrast with previous transcription results from our laboratory, which has shown collagen VI to be upregulated in rotator cuff tears [5], indicating yet again the gap between the transcriptome and the proteome. Nevertheless, these significant changes add weight to recent evidence of the importance of the pericellular region, and specifically collagen VI, in tendon matrix assembly and repair [48, 49].

To summarise other findings, two members of the cartilage intermediate layer protein (CILP) family were identified: CILP1 and CILP2, and both had significantly lower abundance in the aged, torn tendon groups. Proteomics studies have previously identified CILP in horse SDFT [25] as well as human patellar tendon [18], but its localisation and function has not been studied to date. Given the important role of small leucine rich proteoglycans (SLRPs) in tendon development, it was surprising that none of the seven members of the family identified here were significantly modified in the torn tendon samples. Moreover, unlike a previous proteome study demonstrating significant differences in collagens and SLRPs between male and female patellar tendon proteome [18], we did not find any significant gender differences, although admittedly the male and female cohorts compared were both from patients with a rotator cuff tear, and thus not representative of normal, healthy supraspinatus.

Several limitations of the study should be acknowledged. Control samples used for this study were supraspinatus biopsies from patients undergoing shoulder stabilisation. Whilst it was ensured that no macroscopic injury was visible, we cannot eliminate the possibility of microscopic damage to tendon fibres during the biopsy process, leading to changes in protein levels. Moreover, because the torn comparison group was significantly older than the control, some of the differences observed may be due to ageing rather than disease. This is a common problem in studies involving human tissue, where tissue from aged but healthy patients is difficult to collect. Several limitation may arise from the use of ultrasound-guided tissue sampling, including the absence of direct inspection of the tendon and the sampling site, which may lead to a sampling error. However, these limitations are largely offset by enabling a much larger number of donors. Although the small size of the biopsy resulted in relatively few cellular proteins, a good quality extraction of the extracellular compartment was achieved, but another limitation is the insoluble nature of collagen in ageing human tendons [23]. Despite using a harsh and lengthy digestion protocol, it is expected that the digestion of the highly cross-linked collagen I was incomplete, and as evidence to this issue, the ratio of collagen III:I was at least one fold higher than previously reported. Moreover, there remains a risk of a less consistent and incomplete digestion compared with young tendon tissue.

The use of elastase digestion as a validation also carries several limitations. Elastase is an enzyme with a broad specificity and digestion of proteins with this enzyme leads to the generation of overlapping peptides (unlike trypsin, which has a very tight specificity and produces identical, non-overlapping peptides from a protein). For this reason, quantitation of protein expression differences is generally performed using trypsin, as evident throughout the literature. Particularly in this dataset, with highly modified proteins, the trypsin data is the one to be considered more reliable. However, as can be seen from our results, the elastase digestion did provide useful information about additional proteins (see see S2 Table), as well as confirmed the differential abundance of several major ECM proteins.

## Conclusions

To conclude, these findings confirm measurable degradation of collagen fibrils and associated proteins in torn supraspinatus tendons, suggesting that a significant loss of organised tendon tissue is associated with tendon disease and ageing. A marked reduction of COMP strengthen the possibility of using this glycoprotein and its peptides as a marker for tendon injury, as previously suggested in a horse model. The inventory also suggests that elastin is not a major component of human supraspinatus tendon, although more evidence is required to confirm this. Finally, we identified a severe modulation of the elastic fibre, fibrillin-rich niche and the pericellular matrix, suggesting further investigation of these structures could help to explain the cellular processes associated with tendon ageing and tendinopathy.

## Supporting information

S1 Appendix MAscot search results for elastin (ELN) in elastase digested rat aorta. Using the same elastase digestion protocol used on human tendon, elastin was identified in rat aorta with good confidence and sequence coverage.
(PDF)

S1 Table The mean fold change of proteins of all compartments which were upregulated in the torn, trypsin digested samples. (*P<0.05 **P<0.01 significantly different from control in post-test).
(TIF)

S2 Table Proteins identified uniquely in the elastase digested samples and their ANOVA p value. (The arrows indicate if the proteins were downregulated (↓) or upregulated (↑) in the torn samples.).
(TIF)

S3 Table The ratios of collagen III to I and of the subunits of collagen I, (COL1A1 and COL1A2) and collagen VI (COL6A1, COL6A2 and COL6A3) in the elastase digested samples. (mean ± SEM, *P<0.05 significantly different from control).
(TIF)

## References

[1] Fallon J, Blevins FT, Vogel K, Trotter J. Functional morphology of the supraspinatus tendon. Journal of orthopaedic research: official publication of the Orthopaedic Research Society. 2002;20:920–926. 10.1016/S0736-0266(02)00032-2 12382954

[2] Kannus P, Kannus P. Structure of the tendon connective tissue. Scand J Med Sci Sports COPYRIGHT C MUNKSGAARD. 2000;10(6):312–320. 10.1034/j.1600-0838.2000.010006312.x 11085557

[3] Grant TM, Thompson MS, Urban J, Yu J. Elastic fibres are broadly distributed in tendon and highly localized around tenocytes. Journal of Anatomy. 2013;222(6):573–579. 10.1111/joa.12048 23587025 PMC3666236

[4] Fenwick SA, Hazleman BL, Riley GP. The vasculature and its role in the damaged and healing tendon. Arthritis Research. 2002;4(4):252–260. 10.1186/ar416 12106496 PMC128932

[5] Thakkar D, Tyler M Grant, Hakimi O, Carr AJ. Distribution and expression of type VI collagen and elastic fibers in human rotator cuff tendon tears. Connective Tissue Research. 2014;55(5–6):397–402. 10.3109/03008207.2014.959119 25166893

[6] Yamaguchi K, Ditsios K, Middleton WD, Hildebolt CF, Galatz LM, Teefey SA. The Demographic and Morphological Features of Rotator Cuff Disease: A Comparison of Asymptomatic and Symptomatic Shoulders. The Journal of Bone and Joint Surgery. 2006;88(8):1699–1704. 10.2106/JBJS.E.00835 16882890

[7] Bongers PM. The cost of shoulder pain at work. British Medical Journal. 2001;322(7278):64–65. 10.1136/bmj.322.7278.64 11154606 PMC1119373

[8] Nho SJ, Yadav H, Shindle MK, Macgillivray JD. Rotator cuff degeneration: etiology and pathogenesis. The American journal of sports medicine. 2008;36(5):987–993. 18413681 10.1177/0363546508317344

[9] Chaudhury S, Xia Z, Thakkar D, Hakimi O, Carr AJ. Gene expression profiles of changes underlying different-sized human rotator cuff tendon tears. Journal of Shoulder and Elbow Surgery. 2016;. 10.1016/j.jse.2016.02.037 27131575

[10] Tilley JMR, Murphy RJ, Chaudhury S, Czernuszka JT, Carr AJ. Effect of tear size, corticosteroids and subacromial decompression surgery on the hierarchical structural properties of torn supraspinatus tendons. Bone & joint research. 2014;3(8):252–61. 10.1302/2046-3758.38.2000251 25106417 PMC4127658

[11] Goodier HCJ, Carr AJ, Snelling SJB, Roche L, Wheway K, Watkins B, et al Comparison of transforming growth factor beta expression in healthy and diseased human tendon. Arthritis Research & Therapy. 2016;18(1):48 10.1186/s13075-016-0947-8 26883016 PMC4756520

[12] Sejersen MHJ, Frost P, Hansen TB, Deutch SR, Svendsen SW. Proteomics perspectives in rotator cuff research a systematic review of gene expression and protein composition in human tendinopathy. PLoS ONE. 2015;10(4):1–26. 10.1371/journal.pone.0119974 PMC4400011 25879758

[13] Murphy RJ, Floyd Dean BJ, Wheway K, Watkins B, Morrey ME, Carr AJ. A Novel Minimally Invasive Ultrasound-Guided Technique to Biopsy Supraspinatus Tendon. Operative Techniques in Orthopaedics. 2013;23(2):56–62. 10.1053/j.oto.2013.05.003

[14] Dean BJF, Snelling SJB, Dakin SG, Murphy RJ, Javaid MK, Carr AJ. Differences in glutamate receptors and inflammatory cell numbers are associated with the resolution of pain in human rotator cuff tendinopathy. Arthritis research & therapy. 2015;17:176 10.1186/s13075-015-0691-5 26160609 PMC4498529

[15] John Floyd Dean B, Louise Franklin S, Murphy RJ, Javaid MK, Jonathan Carr A. Glucocorticoids induce specific ion-channel-mediated toxicity in human rotator cuff tendon: a mechanism underpinning the ultimately deleterious effect of steroid injection in tendinopathy? Br J Sports Med. 2014;48:1620–1626. 10.1136/bjsports-2013-093178 24677026

[16] Wessel D, Flugge UI. A method for the quantitative recovery of protein in dilute solution in the presence of detergents and lipids. Analytical Biochemistry. 1984;138(1):141–143. 10.1016/0003-2697(84)90782-6 6731838

[17] Adam J, Hatipoglu E, O’Flaherty L, Ternette N, Sahgal N, Lockstone H, et al Renal Cyst Formation in Fh1-Deficient Mice Is Independent of the Hif/Phd Pathway: Roles for Fumarate in KEAP1 Succination and Nrf2 Signaling. Cancer Cell. 2011;20(4):524–537. 10.1016/j.ccr.2011.09.006 22014577 PMC3202623

[18] Little D, Thompson JW, Dubois LG, Ruch DS, Moseley MA, Guilak F. Proteomic differences between male and female anterior cruciate ligament and patellar tendon. PLoS ONE. 2014;9(5). 10.1371/journal.pone.0096526 PMC4018326 24818782

[19] Karonen T, Jeskanen L, Keski-Oja J. Transforming growth factor beta 1 and its latent form binding protein-1 associate with elastic fibres in human dermis: accumulation in actinic damage and absence in anetoderma. The British journal of dermatology. 1997;137(1):51–8. 9274625

[20] Roark EF, Keene DR, Haudenschild CC, Godyna S, Little CD, Argraves WS. The association of human fibulin-1 with elastic fibers: an immunohistological, ultrastructural, and RNA study. The journal of histochemistry and cytochemistry: official journal of the Histochemistry Society. 1995;43(4):401–411. 10.1177/43.4.7534784 7534784

[21] Ritty TM, Roth R, Heuser JE. Tendon cell array isolation reveals a previously unknown fibrillin-2-containing macromolecular assembly. Structure. 2003;11(9):1179–1188. 10.1016/S0969-2126(03)00181-3 12962636

[22] Bank RA, TeKoppele JM, Oostingh G, Hazleman BL, Riley GP. Lysylhydroxylation and non-reducible crosslinking of human supraspinatus tendon collagen: changes with age and in chronic rotator cuff tendinitis. Annals of the rheumatic diseases. 1999;58(1):35–41. 10.1136/ard.58.1.35 10343538 PMC1752756

[23] Riley GP, Harrall RL, Constant CR, Chard MD, Cawston TE, Hazleman BL. Tendon degeneration and chronic shoulder pain: changes in the collagen composition of the human rotator cuff tendons in rotator cuff tendinitis. Annals of the Rheumatic Diseases. 1994;53(6):359–66. 8037494 10.1136/ard.53.6.359 PMC1005350

[24] Chaudhury S, Dicko C, Burgess M, Vollrath F, Carr AJ. Fourier transform infrared spectroscopic analysis of normal and torn rotator-cuff tendons. The Journal of bone and joint surgery British volume. 2011;93(3):370–7. 10.1302/0301-620X.93B3.25470 21357960

[25] Dakin SG, Smith RKW, HeinegArd D, Önnerfjord P, Khabut A, Dudhia J. Proteomic analysis of tendon extracellular matrix reveals disease stage-specific fragmentation and differential cleavage of COMP (cartilage oligomeric matrix protein). Journal of Biological Chemistry. 2014;289(8):4919–4927. 10.1074/jbc.M113.511972 24398684 PMC3931053

[26] Smith RKW, Zunino L, Webbon PM, Heinegård D. The distribution of cartilage oligomeric matrix protein (COMP) in tendon and its variation with tendon site, age and load. Matrix Biology. 1997;16(5):255–271. 10.1016/S0945-053X(97)90014-7 9501326

[27] Södersten F, Hultenby K, Heinegård D, Johnston C, Ekman S. Immunolocalization of collagens (I and III) and cartilage oligomeric matrix protein (COMP) in the normal and injured equine superficial digital flexor tendon. Connective Tissue Research. 2012;54(February 2012):120928103629003. 10.3109/03008207.2012.734879 PMC3545546 23020676

[28] Hamada K, Tomonaga A, Gotoh M, Yamakawa H, Fukuda H. Intrinsic healing capacity and tearing process of torn supraspinatus tendons: In situ hybridization study of *α* 1(I) procollagen mRNA. Journal of Orthopaedic Research. 1997;15(1):24–32. 10.1002/jor.1100150105 9066523

[29] Shindle MK, Chen CCT, Robertson C, DiTullio AE, Paulus MC, Clinton CM, et al Full-thickness supraspinatus tears are associated with more synovial inflammation and tissue degeneration than partial-thickness tears. Journal of Shoulder and Elbow Surgery. 2011;20(6):917–927. 10.1016/j.jse.2011.02.015 21612944 PMC3156316

[30] Shirachi I, Gotoh M, Mitsui Y, Yamada T, Nakama K, Kojima K, et al Collagen production at the edge of ruptured rotator cuff tendon is correlated with postoperative cuff integrity. Arthroscopy—Journal of Arthroscopic and Related Surgery. 2011;27(9):1173–1179. 10.1016/j.arthro.2011.03.078 21752571

[31] Lo IKY, Boorman R, Marchuk L, Hollinshead R, Hart DA, Frank CB. Matrix molecule mRNA levels in the bursa and rotator cuff of patients with full-thickness rotator cuff tears. Arthroscopy—Journal of Arthroscopic and Related Surgery. 2005;21(6):645–651. 10.1016/j.arthro.2005.03.008 15944617

[32] Buckley MR, Evans EB, Matuszewski PE, Chen YL, Satchel LN, Elliott DM, et al Distributions of types I, II and III collagen by region in the human supraspinatus tendon. Connective tissue research. 2013;54(6):374–9. 10.3109/03008207.2013.847096 24088220 PMC6056177

[33] Gibson MA, Finnis ML, Kumaratilake JS, Cleary EG. Microfibril-associated glycoprotein-2 (MAGP-2) is specifically associated with fibrillin-containing microfibrils but exhibits more restricted patterns of tissue localization and developmental expression than its structural relative MAGP-1. The journal of histochemistry and cytochemistry: official journal of the Histochemistry Society. 1998;46(8):871–886. 10.1177/002215549804600802 9671438

[34] Penner AS, Rock MJ, Kielty CM, Michael Shipley J. Microfibril-associated glycoprotein-2 interacts with fibrillin-1 and fibrillin-2 suggesting a role for MAGP-2 in elastic fiber assembly. Journal of Biological Chemistry. 2002;277(38):35044–35049. 10.1074/jbc.M206363200 12122015

[35] Sengle G, Sakai LY. The fibrillin microfibril scaffold: A niche for growth factors and mechanosensation? Matrix Biology. 2015;47:3–12. 10.1016/j.matbio.2015.05.002 25957947

[36] Kobayashi N, Kostka G, Garbe JHO, Keene DR, Bachinger HP, Hanisch FG, et al A Comparative Analysis of the Fibulin Protein Family: biochemical characterization, binding interactions and tissue localization. J Biol Chem. 2007;282(16):11805–11816. 10.1074/jbc.M611029200 17324935

[37] Ritty TM, Ditsios K, Starcher BC. Distribution of the elastic fiber and associated proteins in flexor tendon reflects function. Anatomical Record. 2002;268(4):430–440. 10.1002/ar.10175 12420291

[38] Sugawara Y, Sawada T, Inoue S, Shibayama K, Yanagisawa T. Immunohistochemical localization of elastin, fibrillins and microfibril-associated glycoprotein-1 in the developing periodontal ligament of the rat molar. Journal of Periodontal Research. 2010;45(1):52–59. 10.1111/j.1600-0765.2008.01196.x 19602118

[39] Thomopoulos S, Hattersley G, Rosen V, Mertens M, Galatz L, Williams GR, et al The localized expression of extracellular matrix components in healing tendon insertion sites: an in situ hybridization study. Journal of orthopaedic research: official publication of the Orthopaedic Research Society. 2002;20(3):454–463. 10.1016/S0736-0266(01)00144-9 12038618

[40] Thorpe CT, Karunaseelan KJ, Ng Chieng Hin J, Riley GP, Birch HL, Clegg PD, et al Distribution of proteins within different compartments of tendon varies according to tendon type. Journal of Anatomy. 2016;. 10.1111/joa.12485 PMC4974547 27113131

[41] Jensen SA, Handford PA. New insights into the structure, assembly and biological roles of 10–12 nm connective tissue microfibrils from fibrillin-1 studies. The Biochemical journal. 2016;473(7):827–838. 10.1042/BJ20151108 27026396

[42] Soedersten F, Ekman S, Niehoff A, Zaucke F, Heinegard D, Heinegard D, et al Ultrastructural immunolocalization of cartilage oligomeric matrix protein (COMP) in the articular cartilage on the equine third carpal bone in trained and untrained horses. Research in Veterinary Science. 2010;88(2):251–257. 10.1016/j.rvsc.2009.07.011 19716571

[43] Keene DR, Engvall E, Glanville RW. Ultrastructure of type VI collagen in human skin and cartilage suggests an anchoring function for this filamentous network. Journal of Cell Biology. 1988;107(5):1995–2006. 10.1083/jcb.107.5.1995 3182942 PMC2115316

[44] Lampe aK, Bushby KMD. Collagen VI related muscle disorders. Journal of medical genetics. 2005;42(9):673–85. 10.1136/jmg.2002.002311 16141002 PMC1736127

[45] Lamande S, Sigalas E, Pan Tc, Chu Ml, Dziadek M, Timpl R, et al The Role of the a3(VI) Chain in Collagen VI Assembly. The Journal of Biological Chemistry. 1998;273(13):7423–7430. 10.1074/jbc.273.13.7423 9516440

[46] Hatamochi A, Aumailley M, Mauch C, Chu ML, Timpl R, Krieg T. Regulation of collagen VI expression in fibroblasts. Effects of cell density, cell-matrix interactions, and chemical transformation. Journal of Biological Chemistry. 1989;264(6):3494–3499. 2914960

[47] Thorpe CT, Peffers MJ, Simpson D, Halliwell E, Screen HRC, Clegg PD. Anatomical heterogeneity of tendon: Fascicular and interfascicular tendon compartments have distinct proteomic composition. Scientific reports. 2016;6(October 2015):20455 10.1038/srep20455 26842662 PMC4740843

[48] Sardone F, Santi S, Tagliavini F, Traina F, Merlini L, Squarzoni S, et al Collagen VI-NG2 axis in human tendon fibroblasts under conditions mimicking injury response. Matrix Biology. 2015;. 10.1016/j.matbio.2016.02.012 26944560

[49] Izu Y, Ansorge HL, Zhang G, Soslowsky LJ, Bonaldo P, Chu ML, et al Dysfunctional tendon collagen fibrillogenesis in collagen VI null mice. Matrix Biology. 2011;30(1):53–61. 10.1016/j.matbio.2010.10.001 20951202 PMC3778658

