# Supplementary material for: A quantitative label-free analysis of the extracellular proteome of human supraspinatus tendon reveals damage to the pericellular and elastic fibre niches in torn and aged tissue
Source: PLoS One. 2017 May 18;12(5):e0177656. doi: 10.1371/journal.pone.0177656 (PMC5436668; doi:10.1371/journal.pone.0177656)
Supplement: S1 Appendix — Using the same elastase digestion protocol used on human tendon, elastin was identified in rat aorta with good confidence and sequence coverage. (PDF) [file pone.0177656.s001.pdf]

# MASCOT Search Results

## Protein View: sp|Q99372-2|ELN_RAT

### Isoform 2 of Elastin OS=Rattus norvegicus GN=Eln

| | |
|---|---|
| **Database:** | UPR_RattusNorvegicus |
| **Score:** | 2093 |
| **Nominal mass ($M_r$):** | 69230 |
| **Calculated pI:** | 10.44 |

Sequence similarity is available as an NCBI BLAST search of sp|Q99372-2|ELN_RAT against nr.

### Search parameters

| | |
|---|---|
| **Enzyme:** | No enzyme cleavage specificity. |
| **Fixed modifications:** | Carbamidomethyl (C) |
| **Variable modifications:** | Oxidation (P), Oxidation (K), Lys->Allysine (K) |

### Protein sequence coverage: 57%

Matched peptides shown in ***bold red***.

```
  1 MAGLTAAVPQ PGVLLILLLN LLHPAQPGGV PGAVPGGVPG GLPGGVPGGV
 51 YYPGAGIGGG LGGGALGPGG KPPKPGAGLL GAFGAGPGGL GGAGPGAGLS
101 YASRPGGVLV PGGGAGAAAA YKAAAKAGAG LGGIGGVPGG VGVGGVPGAV
151 GVGGVPGAVG GIGGIGGLGV STGAVVPQLG AGVGAGGKPG KVPGVGLPGV
201 YPGGVLPGTG ARFPGVGVLP GVPTGTGVKA KVPGGGGGAF SGIPGVGPFG
251 GQQPGVPLGY PIKAPKLPAG AGGGGVLPGV GGGGIPGGAG AIPGIGGITG
301 AGTPAAAAAA KAAAKAAKYG AAGGLVPGGP GVRVPGAGIP GVGIPGVGGI
351 PGVGGIPGVG GIPGVGGPGI GGPGIVGGPG AVSPAAAAKA AAKAAKYGAR
401 GGVGIPTYGV GAGGFPGYGV GAGAGLGGAS QAAAAAAAAK AAKYGAGGAG
451 TLGGLVPGAV PGALPGAVPG ALPGAVPGAL PGAVPGVPGT GGVPGAGTPA
501 AAAAAAAAKA AAKAGQYGLG PGVGGVPGGV GVGGLPGGVG PGGVTGIGTG
551 PGTGLVPGDL GGAGTPAAAK SAAKAAAKAQ YRAAAGLGAG VPGLGVGAGV
601 PGFGAGAGGF GAGAGVPGFG AGAVPGSLAA SKAAKYGAAG GLGGPGGLGG
651 PGGLGGPGGF GGPGGLGGVP GGVAGGAPAA AAAAKAAAKA AQYGLGGAGG
701 LGAGGLGAGG LGAGGLGAGG LGAGGLGAGG VIPGAVGLGG VSPAAAAKAA
751 KYGAAGLGGV LGARPFPGGG VAARPGFGLS PIYPGGGAGG LGVGGKPPKP
801 YGGALGALGY QGGGCFGKSC GRKRK
```

Unformatted sequence string: 825 residues (for pasting into other applications).

Sort peptides by ● Residue Number ○ Increasing Mass ○ Decreasing Mass

| Query | Start – End | Observed | Mr(expt) | Mr(calc) | ppm | M | Score | Expect | Rank | U | Peptide |
|---|---|---|---|---|---|---|---|---|---|---|---|
| 7427 | 33 – 50 | 730.4008 | 1458.7870 | 1458.7831 | 2.62 | 0 | 26 | 0.12 | 1 | U | G.AVPGGVPGGLPGGVPGGV.Y + Oxidation (P) |
| 3914 | 37 – 50 | 560.3115 | 1118.6084 | 1118.6085 | -0.078 | 0 | 65 | 0.00081 | 1 | U | G.GVPGGLPGGVPGGV.Y |
| 3916 | 37 – 50 | 560.3120 | 1118.6093 | 1118.6085 | 0.80 | 0 | 24 | 0.18 | 1 | U | G.GVPGGLPGGVPGGV.Y |
| 9484 | 79 – 100 | 877.4682 | 1752.9218 | 1752.9159 | 3.39 | 0 | 78 | 2.1e-06 | 1 | U | G.LLGAFGAGPGGLGGAGPGAGLS.Y |
| 6653 | 81 – 98 | 664.3215 | 1326.6285 | 1326.6317 | -2.40 | 0 | 22 | 0.099 | 1 | U | L.GAFGAGPGGLGGAGPGAG.L |
| 7853 | 81 – 100 | 764.3790 | 1526.7434 | 1526.7478 | -2.89 | 0 | 92 | 9.9e-08 | 1 | U | L.GAFGAGPGGLGGAGPGAGLS.Y |
| 7854 | 81 – 100 | 764.3793 | 1526.7440 | 1526.7478 | -2.49 | 0 | 32 | 0.03 | 1 | U | L.GAFGAGPGGLGGAGPGAGLS.Y |
| 7855 | 81 – 100 | 764.3795 | 1526.7444 | 1526.7478 | -2.24 | 0 | 66 | 7.2e-05 | 1 | U | L.GAFGAGPGGLGGAGPGAGLS.Y |
| 7856 | 81 – 100 | 764.3799 | 1526.7453 | 1526.7478 | -1.61 | 0 | 88 | 1.5e-06 | 1 | U | L.GAFGAGPGGLGGAGPGAGLS.Y |
| 7857 | 81 – 100 | 764.3806 | 1526.7466 | 1526.7478 | -0.81 | 0 | 48 | 0.0004 | 1 | U | L.GAFGAGPGGLGGAGPGAGLS.Y |
| 7858 | 81 – 100 | 764.3811 | 1526.7477 | 1526.7478 | -0.086 | 0 | 87 | 7.9e-06 | 1 | U | L.GAFGAGPGGLGGAGPGAGLS.Y |
| 7859 | 81 – 100 | 764.3816 | 1526.7486 | 1526.7478 | 0.55 | 0 | 109 | 6.1e-08 | 1 | U | L.GAFGAGPGGLGGAGPGAGLS.Y |
| 7860 | 81 – 100 | 764.3821 | 1526.7496 | 1526.7478 | 1.19 | 0 | 27 | 0.065 | 1 | U | L.GAFGAGPGGLGGAGPGAGLS.Y |
| 7861 | 81 – 100 | 764.3827 | 1526.7508 | 1526.7478 | 1.99 | 0 | 57 | 0.00028 | 1 | U | L.GAFGAGPGGLGGAGPGAGLS.Y |
| 7862 | 81 – 100 | 764.3829 | 1526.7512 | 1526.7478 | 2.23 | 0 | 118 | 8.2e-09 | 1 | U | L.GAFGAGPGGLGGAGPGAGLS.Y |
| 6552 | 83 – 99 | 656.8343 | 1311.6540 | 1311.6572 | -2.40 | 0 | 27 | 0.12 | 2 | U | A.FGAGPGGLGGAGPGAGL.S |
| 7095 | 83 – 100 | 700.3506 | 1398.6866 | 1398.6892 | -1.85 | 0 | 41 | 0.069 | 1 | U | A.FGAGPGGLGGAGPGAGLS.Y |
| 7096 | 83 – 100 | 700.3507 | 1398.6869 | 1398.6892 | -1.68 | 0 | 25 | 0.26 | 1 | U | A.FGAGPGGLGGAGPGAGLS.Y |
| 7097 | 83 – 100 | 700.3511 | 1398.6877 | 1398.6892 | -1.07 | 0 | 57 | 0.0016 | 1 | U | A.FGAGPGGLGGAGPGAGLS.Y |
| 7098 | 83 – 100 | 700.3516 | 1398.6886 | 1398.6892 | -0.46 | 0 | 35 | 0.22 | 1 | U | A.FGAGPGGLGGAGPGAGLS.Y |
| 7100 | 83 – 100 | 700.3520 | 1398.6894 | 1398.6892 | 0.16 | 0 | 64 | 0.00018 | 1 | U | A.FGAGPGGLGGAGPGAGLS.Y |
| 7101 | 83 – 100 | 700.3522 | 1398.6898 | 1398.6892 | 0.42 | 0 | 35 | 0.023 | 1 | U | A.FGAGPGGLGGAGPGAGLS.Y |
| 7102 | 83 – 100 | 700.3522 | 1398.6899 | 1398.6892 | 0.51 | 0 | 20 | 0.69 | 1 | U | A.FGAGPGGLGGAGPGAGLS.Y |
| 7620 | 101 – 117 | 743.3932 | 1484.7718 | 1484.7736 | -1.19 | 0 | 35 | 0.053 | 1 | U | S.YASRPGGVLVPGGGAGA.A |
| 7671 | 101 – 117 | 751.3923 | 1500.7700 | 1500.7685 | 0.99 | 0 | 33 | 0.34 | 1 | U | S.YASRPGGVLVPGGGAGA.A + Oxidation (P) |
| 8049 | 101 – 118 | 778.9129 | 1555.8113 | 1555.8107 | 0.36 | 0 | 39 | 0.0061 | 1 | U | S.YASRPGGVLVPGGGAGAA.A |
| 8471 | 101 – 118 | 786.9116 | 1571.8087 | 1571.8056 | 1.95 | 0 | 40 | 0.0068 | 1 | U | S.YASRPGGVLVPGGGAGAA.A + Oxidation (P) |
| 5714 | 103 – 117 | 626.3439 | 1250.6732 | 1250.6732 | 0.028 | 0 | 49 | 0.0023 | 1 | U | A.SRPGGVLVPGGGAGA.A |
| 5715 | 103 – 117 | 626.3441 | 1250.6737 | 1250.6732 | 0.42 | 0 | 59 | 0.003 | 1 | U | A.SRPGGVLVPGGGAGA.A |
| 5716 | 103 – 117 | 626.3441 | 1250.6737 | 1250.6732 | 0.42 | 0 | 52 | 0.0008 | 1 | U | A.SRPGGVLVPGGGAGA.A |
| 5717 | 103 – 117 | 626.3448 | 1250.6750 | 1250.6732 | 1.49 | 0 | 38 | 0.026 | 1 | U | A.SRPGGVLVPGGGAGA.A |
| 5720 | 103 – 117 | 626.3458 | 1250.6771 | 1250.6732 | 3.15 | 0 | 37 | 0.3 | 1 | U | A.SRPGGVLVPGGGAGA.A |
| 5721 | 103 – 117 | 626.3464 | 1250.6782 | 1250.6732 | 4.03 | 0 | 35 | 0.21 | 1 | U | A.SRPGGVLVPGGGAGA.A |
| 5926 | 103 – 117 | 634.3423 | 1266.6701 | 1266.6681 | 1.63 | 0 | 62 | 0.0006 | 1 | U | A.SRPGGVLVPGGGAGA.A + Oxidation (P) |
| 5927 | 103 – 117 | 634.3439 | 1266.6733 | 1266.6681 | 4.14 | 0 | 51 | 0.0016 | 1 | U | A.SRPGGVLVPGGGAGA.A + Oxidation (P) |
| 6616 | 103 – 118 | 661.8624 | 1321.7103 | 1321.7103 | 0.027 | 0 | 23 | 0.24 | 1 | U | A.SRPGGVLVPGGGAGAA.A |
| 6617 | 103 – 118 | 661.8625 | 1321.7105 | 1321.7103 | 0.21 | 0 | 26 | 0.062 | 1 | U | A.SRPGGVLVPGGGAGAA.A |
| 6618 | 103 – 118 | 661.8626 | 1321.7107 | 1321.7103 | 0.30 | 0 | 47 | 0.0063 | 1 | U | A.SRPGGVLVPGGGAGAA.A |
| 6619 | 103 – 118 | 661.8629 | 1321.7112 | 1321.7103 | 0.67 | 0 | 51 | 0.0036 | 1 | U | A.SRPGGVLVPGGGAGAA.A |
| 6620 | 103 – 118 | 661.8629 | 1321.7112 | 1321.7103 | 0.67 | 0 | 46 | 0.0092 | 1 | U | A.SRPGGVLVPGGGAGAA.A |
| 6621 | 103 – 118 | 661.8632 | 1321.7118 | 1321.7103 | 1.13 | 0 | 52 | 0.0025 | 1 | U | A.SRPGGVLVPGGGAGAA.A |

| Query | Start – End | Observed | Mr(expt) | Mr(calc) | ppm | M | Score | Expect | Rank | U | Peptide |
|---|---|---|---|---|---|---|---|---|---|---|---|
| 6622 | 103 – 118 | 661.8640 | 1321.7135 | 1321.7103 | 2.43 | 0 | 31 | 0.11 | 1 | U | A.SRPGGVLVPGGGAGAA.A |
| 6698 | 103 – 118 | 669.8608 | 1337.7070 | 1337.7052 | 1.36 | 0 | 47 | 0.008 | 1 | U | A.SRPGGVLVPGGGAGAA.A + Oxidation (P) |
| 6699 | 103 – 118 | 669.8611 | 1337.7076 | 1337.7052 | 1.82 | 0 | 58 | 0.00027 | 1 | U | A.SRPGGVLVPGGGAGAA.A + Oxidation (P) |
| 6700 | 103 – 118 | 669.8614 | 1337.7082 | 1337.7052 | 2.27 | 0 | 46 | 0.0051 | 1 | U | A.SRPGGVLVPGGGAGAA.A + Oxidation (P) |
| 5463 | 128 – 143 | 612.3397 | 1222.6649 | 1222.6670 | -1.76 | 0 | 22 | 1.3 | 2 | U | A.GAGLGGIGGVPGGVGV.G |
| 9748 | 128 – 150 | 880.9897 | 1759.9648 | 1759.9582 | 3.78 | 0 | 20 | 0.12 | 1 | U | A.GAGLGGIGGVPGGVGVGGVPGAV.G |
| 10525 | 128 – 152 | 967.0302 | 1932.0457 | 1932.0430 | 1.44 | 0 | 31 | 0.068 | 1 | U | A.GAGLGGIGGVPGGVGVGGVPGAVGV.G + Oxidation (P) |
| 9314 | 149 – 170 | 860.4915 | 1718.9684 | 1718.9680 | 0.22 | 0 | 34 | 0.0059 | 1 | U | G.AVGVGGVPGAVGGIGGIGGLGV.S |
| 9315 | 149 – 170 | 860.4917 | 1718.9688 | 1718.9680 | 0.50 | 0 | 64 | 3.6e-05 | 1 | U | G.AVGVGGVPGAVGGIGGIGGLGV.S |
| 9390 | 149 – 170 | 868.4871 | 1734.9596 | 1734.9629 | -1.92 | 0 | 63 | 6.6e-05 | 1 | U | G.AVGVGGVPGAVGGIGGIGGLGV.S + Oxidation (P) |
| 9391 | 149 – 170 | 868.4883 | 1734.9620 | 1734.9629 | -0.52 | 0 | 32 | 0.068 | 1 | U | G.AVGVGGVPGAVGGIGGIGGLGV.S + Oxidation (P) |
| 9392 | 149 – 170 | 868.4890 | 1734.9635 | 1734.9629 | 0.33 | 0 | 50 | 0.0011 | 1 | U | G.AVGVGGVPGAVGGIGGIGGLGV.S + Oxidation (P) |
| 7993 | 151 – 170 | 775.4387 | 1548.8628 | 1548.8625 | 0.20 | 0 | 45 | 0.0018 | 1 | U | V.GVGGVPGAVGGIGGIGGLGV.S |
| 7994 | 151 – 170 | 775.4388 | 1548.8630 | 1548.8625 | 0.36 | 0 | 86 | 8e-07 | 1 | U | V.GVGGVPGAVGGIGGIGGLGV.S |
| 7995 | 151 – 170 | 775.4388 | 1548.8631 | 1548.8625 | 0.44 | 0 | 39 | 0.024 | 1 | U | V.GVGGVPGAVGGIGGIGGLGV.S |
| 7997 | 151 – 170 | 775.4392 | 1548.8639 | 1548.8625 | 0.91 | 0 | 91 | 2.1e-06 | 1 | U | V.GVGGVPGAVGGIGGIGGLGV.S |
| 8000 | 151 – 170 | 775.4401 | 1548.8656 | 1548.8625 | 2.01 | 0 | 31 | 0.081 | 1 | U | V.GVGGVPGAVGGIGGIGGLGV.S |
| 8142 | 151 – 170 | 783.4376 | 1564.8607 | 1564.8574 | 2.12 | 0 | 66 | 0.00035 | 1 | U | V.GVGGVPGAVGGIGGIGGLGV.S + Oxidation (P) |
| 7065 | 153 – 170 | 697.3937 | 1392.7728 | 1392.7726 | 0.17 | 0 | 77 | 2.4e-05 | 1 | U | V.GGVPGAVGGIGGIGGLGV.S |
| 7066 | 153 – 170 | 697.3940 | 1392.7735 | 1392.7726 | 0.69 | 0 | 91 | 2e-06 | 1 | U | V.GGVPGAVGGIGGIGGLGV.S |
| 7068 | 153 – 170 | 697.3943 | 1392.7741 | 1392.7726 | 1.13 | 0 | 56 | 0.00022 | 1 | U | V.GGVPGAVGGIGGIGGLGV.S |
| 7069 | 153 – 170 | 697.3944 | 1392.7743 | 1392.7726 | 1.22 | 0 | 68 | 1.5e-05 | 1 | U | V.GGVPGAVGGIGGIGGLGV.S |
| 7071 | 153 – 170 | 697.3950 | 1392.7754 | 1392.7726 | 2.01 | 0 | 33 | 0.009 | 1 | U | V.GGVPGAVGGIGGIGGLGV.S |
| 7072 | 153 – 170 | 697.3951 | 1392.7756 | 1392.7726 | 2.18 | 0 | 55 | 0.00056 | 1 | U | V.GGVPGAVGGIGGIGGLGV.S |
| 7155 | 153 – 170 | 705.3910 | 1408.7674 | 1408.7675 | -0.040 | 0 | 40 | 0.0073 | 1 | U | V.GGVPGAVGGIGGIGGLGV.S + Oxidation (P) |
| 7156 | 153 – 170 | 705.3910 | 1408.7674 | 1408.7675 | -0.040 | 0 | 61 | 8e-05 | 1 | U | V.GGVPGAVGGIGGIGGLGV.S + Oxidation (P) |
| 7157 | 153 – 170 | 705.3915 | 1408.7684 | 1408.7675 | 0.65 | 0 | 69 | 2.6e-05 | 1 | U | V.GGVPGAVGGIGGIGGLGV.S + Oxidation (P) |
| 7158 | 153 – 170 | 705.3916 | 1408.7687 | 1408.7675 | 0.83 | 0 | 40 | 0.013 | 1 | U | V.GGVPGAVGGIGGIGGLGV.S + Oxidation (P) |
| 7159 | 153 – 170 | 705.3918 | 1408.7690 | 1408.7675 | 1.09 | 0 | 41 | 0.0048 | 1 | U | V.GGVPGAVGGIGGIGGLGV.S + Oxidation (P) |
| 2889 | 158 – 170 | 513.7999 | 1025.5853 | 1025.5870 | -1.63 | 0 | 52 | 0.0041 | 1 | U | G.AVGGIGGIGGLGV.S |
| 2890 | 158 – 170 | 513.8002 | 1025.5859 | 1025.5870 | -1.04 | 0 | 26 | 0.24 | 1 | U | G.AVGGIGGIGGLGV.S |
| 2891 | 158 – 170 | 513.8004 | 1025.5862 | 1025.5870 | -0.80 | 0 | 33 | 0.057 | 1 | U | G.AVGGIGGIGGLGV.S |
| 4527 | 171 – 183 | 578.3230 | 1154.6314 | 1154.6296 | 1.63 | 0 | 29 | 0.52 | 1 | U | V.STGAVVPQLGAGV.G |
| 2216 | 173 – 183 | 484.2834 | 966.5523 | 966.5498 | 2.58 | 0 | 29 | 0.098 | 1 | U | T.GAVVPQLGAGV.G |
| 3661 | 173 – 185 | 548.3121 | 1094.6097 | 1094.6084 | 1.18 | 0 | 21 | 0.16 | 1 | U | T.GAVVPQLGAGVGA.G |
| 499 | 174 – 181 | 377.7271 | 753.4397 | 753.4385 | 1.65 | 0 | 34 | 0.28 | 1 | U | G.AVVPQLGA.G |
| 1444 | 174 – 183 | 455.7703 | 909.5260 | 909.5284 | -2.58 | 0 | 63 | 0.00029 | 1 | U | G.AVVPQLGAGV.G |
| 1446 | 174 – 183 | 455.7719 | 909.5292 | 909.5284 | 0.91 | 0 | 38 | 0.022 | 1 | U | G.AVVPQLGAGV.G |
| 1447 | 174 – 183 | 455.7722 | 909.5299 | 909.5284 | 1.65 | 0 | 27 | 0.18 | 1 | U | G.AVVPQLGAGV.G |
| 1448 | 174 – 183 | 455.7730 | 909.5314 | 909.5284 | 3.33 | 0 | 27 | 0.22 | 1 | U | G.AVVPQLGAGV.G |
| 3028 | 174 – 185 | 519.8007 | 1037.5869 | 1037.5870 | -0.069 | 0 | 25 | 0.45 | 1 | U | G.AVVPQLGAGVGA.G |
| 1020 | 175 – 183 | 420.2519 | 838.4892 | 838.4913 | -2.50 | 0 | 23 | 0.13 | 1 | U | A.VVPQLGAGV.G |
| 7831 | 209 – 224 | 762.9279 | 1523.8412 | 1523.8461 | -3.21 | 0 | 24 | 0.17 | 1 | U | G.TGARFPGVGVLPGVPT.G |
| 7832 | 209 – 224 | 762.9283 | 1523.8421 | 1523.8461 | -2.57 | 0 | 36 | 0.017 | 1 | U | G.TGARFPGVGVLPGVPT.G |
| 6921 | 211 – 224 | 683.8937 | 1365.7729 | 1365.7769 | -2.91 | 0 | 39 | 0.028 | 1 | U | G.ARFPGVGVLPGVPT.G |
| 6922 | 211 – 224 | 683.8942 | 1365.7738 | 1365.7769 | -2.29 | 0 | 23 | 0.16 | 1 | U | G.ARFPGVGVLPGVPT.G |
| 6923 | 211 – 224 | 683.8945 | 1365.7745 | 1365.7769 | -1.75 | 0 | 34 | 0.039 | 1 | U | G.ARFPGVGVLPGVPT.G |
| 6383 | 212 – 224 | 648.3751 | 1294.7356 | 1294.7398 | -3.26 | 0 | 39 | 0.019 | 1 | U | A.RFPGVGVLPGVPT.G |
| 6387 | 212 – 224 | 648.3752 | 1294.7358 | 1294.7398 | -3.07 | 0 | 40 | 0.049 | 1 | U | A.RFPGVGVLPGVPT.G |
| 6388 | 212 – 224 | 648.3752 | 1294.7359 | 1294.7398 | -2.98 | 0 | 55 | 0.0032 | 1 | U | A.RFPGVGVLPGVPT.G |
| 6390 | 212 – 224 | 648.3755 | 1294.7365 | 1294.7398 | -2.51 | 0 | 26 | 0.31 | 1 | U | A.RFPGVGVLPGVPT.G |
| 6394 | 212 – 224 | 648.3758 | 1294.7370 | 1294.7398 | -2.13 | 0 | 40 | 0.019 | 1 | U | A.RFPGVGVLPGVPT.G |
| 6395 | 212 – 224 | 648.3759 | 1294.7373 | 1294.7398 | -1.94 | 0 | 43 | 0.035 | 1 | U | A.RFPGVGVLPGVPT.G |
| 6397 | 212 – 224 | 648.3761 | 1294.7376 | 1294.7398 | -1.66 | 0 | 24 | 0.18 | 1 | U | A.RFPGVGVLPGVPT.G |
| 6398 | 212 – 224 | 648.3761 | 1294.7376 | 1294.7398 | -1.66 | 0 | 39 | 0.019 | 1 | U | A.RFPGVGVLPGVPT.G |
| 6405 | 212 – 224 | 648.3766 | 1294.7387 | 1294.7398 | -0.81 | 0 | 25 | 0.19 | 2 | U | A.RFPGVGVLPGVPT.G |
| 6406 | 212 – 224 | 648.3767 | 1294.7389 | 1294.7398 | -0.72 | 0 | 21 | 0.65 | 2 | U | A.RFPGVGVLPGVPT.G |
| 6409 | 212 – 224 | 648.3771 | 1294.7396 | 1294.7398 | -0.15 | 0 | 23 | 0.34 | 1 | U | A.RFPGVGVLPGVPT.G |
| 6411 | 212 – 224 | 648.3773 | 1294.7400 | 1294.7398 | 0.13 | 0 | 25 | 0.38 | 1 | U | A.RFPGVGVLPGVPT.G |
| 6417 | 212 – 224 | 648.3776 | 1294.7406 | 1294.7398 | 0.60 | 0 | 24 | 0.31 | 1 | U | A.RFPGVGVLPGVPT.G |
| 6421 | 212 – 224 | 648.3780 | 1294.7414 | 1294.7398 | 1.27 | 0 | 20 | 0.31 | 1 | U | A.RFPGVGVLPGVPT.G |
| 6423 | 212 – 224 | 648.3782 | 1294.7419 | 1294.7398 | 1.64 | 0 | 33 | 0.1 | 1 | U | A.RFPGVGVLPGVPT.G |
| 10144 | 240 – 258 | 913.9766 | 1825.9387 | 1825.9363 | 1.29 | 0 | 43 | 0.0013 | 1 | U | A.FSGIPGVGPFGGQQPGVPL.G + Oxidation (P) |
| 8601 | 242 – 258 | 796.9250 | 1591.8354 | 1591.8359 | -0.29 | 0 | 26 | 0.14 | 1 | U | S.GIPGVGPFGGQQPGVPL.G + Oxidation (P) |
| 8602 | 242 – 258 | 796.9251 | 1591.8357 | 1591.8359 | -0.14 | 0 | 44 | 0.0025 | 1 | U | S.GIPGVGPFGGQQPGVPL.G + Oxidation (P) |
| 8605 | 242 – 258 | 796.9255 | 1591.8365 | 1591.8359 | 0.40 | 0 | 37 | 0.039 | 1 | U | S.GIPGVGPFGGQQPGVPL.G + Oxidation (P) |
| 8607 | 242 – 258 | 796.9257 | 1591.8369 | 1591.8359 | 0.63 | 0 | 62 | 0.00014 | 1 | U | S.GIPGVGPFGGQQPGVPL.G + Oxidation (P) |
| 8627 | 280 – 299 | 797.4328 | 1592.8510 | 1592.8523 | -0.76 | 0 | 38 | 0.034 | 1 | U | G.VGGGGIPGGAGAIPGIGGIT.G + Oxidation (P) |
| 8719 | 280 – 299 | 805.4313 | 1608.8480 | 1608.8472 | 0.51 | 0 | 35 | 0.027 | 1 | U | G.VGGGGIPGGAGAIPGIGGIT.G + 2 Oxidation (P) |
| 9180 | 280 – 301 | 853.4673 | 1704.9201 | 1704.9159 | 2.47 | 0 | 24 | 0.098 | 1 | U | G.VGGGGIPGGAGAIPGIGGITGA.G |
| 9324 | 280 – 301 | 861.4605 | 1720.9065 | 1720.9108 | -2.54 | 0 | 25 | 0.043 | 1 | U | G.VGGGGIPGGAGAIPGIGGITGA.G + Oxidation (P) |
| 9325 | 280 – 301 | 861.4610 | 1720.9074 | 1720.9108 | -1.97 | 0 | 23 | 0.22 | 1 | U | G.VGGGGIPGGAGAIPGIGGITGA.G + Oxidation (P) |
| 10886 | 280 – 305 | 1032.5426 | 2063.0707 | 2063.0648 | 2.84 | 0 | 50 | 0.0027 | 1 | U | G.VGGGGIPGGAGAIPGIGGITGAGTPA.A + 2 Oxidation (P) |
| 6531 | 290 – 305 | 655.3605 | 1308.7064 | 1308.7038 | 2.00 | 0 | 25 | 0.11 | 1 | U | A.GAIPGIGGITGAGTPA.A |
| 6532 | 290 – 305 | 655.3613 | 1308.7080 | 1308.7038 | 3.21 | 0 | 26 | 0.063 | 1 | U | A.GAIPGIGGITGAGTPA.A |
| 6299 | 323 – 337 | 645.3711 | 1288.7276 | 1288.7252 | 1.89 | 0 | 23 | 0.078 | 1 | U | A.GGLVPGGPGVRVPGA.G |
| 6732 | 408 – 422 | 672.8140 | 1343.6135 | 1343.6147 | -0.86 | 0 | 36 | 0.07 | 1 | U | T.YGVGAGGFPGYGVGA.G + Oxidation (P) |
| 6734 | 408 – 422 | 672.8142 | 1343.6139 | 1343.6147 | -0.59 | 0 | 21 | 0.63 | 1 | U | T.YGVGAGGFPGYGVGA.G + Oxidation (P) |
| 7515 | 408 – 424 | 736.8448 | 1471.6751 | 1471.6732 | 1.30 | 0 | 41 | 0.0041 | 1 | U | T.YGVGAGGFPGYGVGAGA.G + Oxidation (P) |
| 10154 | 408 – 429 | 914.4352 | 1826.8559 | 1826.8588 | -1.58 | 0 | 28 | 0.069 | 1 | U | T.YGVGAGGFPGYGVGAGAGLGGA.S + Oxidation (P) |
| 1136 | 423 – 433 | 430.2168 | 858.4190 | 858.4195 | -0.58 | 0 | 42 | 0.062 | 1 | U | A.GAGLGGASQAA.A |

| Query | Start – End | Observed | Mr(expt) | Mr(calc) | ppm | M | Score | Expect | Rank | U | Peptide |
|---|---|---|---|---|---|---|---|---|---|---|---|
| 2633 | 459 – 470 | 503.2910 | 1004.5674 | 1004.5655 | 1.86 | 0 | 32 | 0.032 | 1 | U | G.AVPGALPGAVPG.A |
| 2634 | 459 – 470 | 503.2912 | 1004.5679 | 1004.5655 | 2.40 | 0 | 41 | 0.0034 | 1 | U | G.AVPGALPGAVPG.A |
| 251 | 463 – 470 | 341.2001 | 680.3856 | 680.3857 | -0.20 | 0 | 20 | 0.99 | 2 | U | G.ALPGAVPG.A |
| 255 | 463 – 470 | 341.2002 | 680.3858 | 680.3857 | 0.16 | 0 | 21 | 1.8 | 3 | U | G.ALPGAVPG.A |
| 256 | 463 – 470 | 341.2002 | 680.3858 | 680.3857 | 0.16 | 0 | 22 | 0.39 | 2 | U | G.ALPGAVPG.A |
| 2796 | 463 – 474 | 510.2981 | 1018.5816 | 1018.5811 | 0.50 | 0 | 32 | 0.047 | 1 | U | G.ALPGAVPGALPG.A |
| 10170 | 479 – 500 | 916.4836 | 1830.9527 | 1830.9476 | 2.79 | 0 | 26 | 0.025 | 1 | U | G.ALPGAVPGVPGTGGVPGAGTPA.A + 2 Oxidation (P) |
| 7431 | 483 – 500 | 731.3887 | 1460.7628 | 1460.7624 | 0.28 | 0 | 35 | 0.012 | 1 | U | G.AVPGVPGTGGVPGAGTPA.A |
| 7432 | 483 – 500 | 731.3898 | 1460.7650 | 1460.7624 | 1.78 | 0 | 41 | 0.0043 | 1 | U | G.AVPGVPGTGGVPGAGTPA.A |
| 7534 | 483 – 500 | 739.3878 | 1476.7610 | 1476.7573 | 2.47 | 0 | 22 | 0.19 | 1 | U | G.AVPGVPGTGGVPGAGTPA.A + Oxidation (P) |
| 7850 | 515 – 532 | 763.9006 | 1525.7867 | 1525.7890 | -1.47 | 0 | 30 | 0.036 | 1 | U | A.GQYGLGPGVGGVPGGVGV.G |
| 7696 | 529 – 547 | 753.9154 | 1505.8163 | 1505.8203 | -2.66 | 0 | 21 | 0.13 | 1 | U | G.GVGVGGLPGGVGPGGVTGI.G |
| 7697 | 529 – 547 | 753.9161 | 1505.8177 | 1505.8203 | -1.69 | 0 | 36 | 0.0084 | 1 | U | G.GVGVGGLPGGVGPGGVTGI.G |
| 4782 | 531 – 545 | 590.8207 | 1179.6268 | 1179.6248 | 1.66 | 0 | 24 | 0.14 | 1 | U | V.GVGGLPGGVGPGGVT.G |
| 6806 | 531 – 547 | 675.8700 | 1349.7254 | 1349.7304 | -3.66 | 0 | 30 | 0.057 | 1 | U | V.GVGGLPGGVGPGGVTGI.G |
| 6914 | 531 – 547 | 683.8712 | 1365.7278 | 1365.7253 | 1.80 | 0 | 34 | 0.016 | 1 | U | V.GVGGLPGGVGPGGVTGI.G + Oxidation (P) |
| 2843 | 533 – 545 | 512.7759 | 1023.5373 | 1023.5350 | 2.32 | 0 | 22 | 0.1 | 1 | U | V.GGLPGGVGPGGVT.G |
| 2844 | 533 – 545 | 512.7760 | 1023.5374 | 1023.5350 | 2.43 | 0 | 21 | 0.46 | 2 | U | V.GGLPGGVGPGGVT.G |
| 4908 | 533 – 547 | 597.8282 | 1193.6418 | 1193.6405 | 1.12 | 0 | 31 | 0.033 | 2 | U | V.GGLPGGVGPGGVTGI.G |
| 5212 | 533 – 547 | 605.8267 | 1209.6388 | 1209.6354 | 2.78 | 0 | 32 | 0.065 | 1 | U | V.GGLPGGVGPGGVTGI.G + Oxidation (P) |
| 8958 | 548 – 567 | 826.4163 | 1650.8181 | 1650.8214 | -1.98 | 0 | 24 | 0.083 | 2 | U | I.GTGPGTGLVPGDLGGAGTPA.A |
| 8962 | 548 – 567 | 826.4176 | 1650.8207 | 1650.8214 | -0.43 | 0 | 44 | 0.0046 | 1 | U | I.GTGPGTGLVPGDLGGAGTPA.A |
| 8965 | 548 – 567 | 826.4182 | 1650.8218 | 1650.8214 | 0.23 | 0 | 29 | 0.074 | 1 | U | I.GTGPGTGLVPGDLGGAGTPA.A |
| 8966 | 548 – 567 | 826.4184 | 1650.8222 | 1650.8214 | 0.53 | 0 | 30 | 0.032 | 1 | U | I.GTGPGTGLVPGDLGGAGTPA.A |
| 8968 | 548 – 567 | 826.4196 | 1650.8247 | 1650.8214 | 2.01 | 0 | 40 | 0.01 | 1 | U | I.GTGPGTGLVPGDLGGAGTPA.A |
| 2212 | 585 – 596 | 484.2810 | 966.5475 | 966.5498 | -2.41 | 0 | 22 | 0.68 | 1 | U | A.AGLGAGVPGLGV.G |
| 4088 | 599 – 612 | 561.2714 | 1120.5283 | 1120.5302 | -1.70 | 0 | 40 | 0.009 | 1 | U | A.GVPGFGAGAGGFGA.G |
| 4089 | 599 – 612 | 561.2714 | 1120.5283 | 1120.5302 | -1.70 | 0 | 33 | 0.064 | 1 | U | A.GVPGFGAGAGGFGA.G |
| 5684 | 599 – 614 | 625.3021 | 1248.5896 | 1248.5888 | 0.64 | 0 | 51 | 0.00057 | 1 | U | A.GVPGFGAGAGGFGAGA.G |
| 7673 | 608 – 626 | 751.8702 | 1501.7259 | 1501.7314 | -3.67 | 0 | 20 | 0.12 | 1 | U | A.GGFGAGAGVPGFGAGAVPG.S |
| 2442 | 615 – 626 | 493.2596 | 984.5046 | 984.5029 | 1.66 | 0 | 28 | 0.51 | 1 | U | A.GVPGFGAGAVPG.S |
| 11585 | 640 – 673 | 1278.6577 | 2555.3009 | 2555.2882 | 4.96 | 0 | 44 | 0.0019 | 1 | U | A.GGLGGPGGLGGPGGLGGPGGFGGPGGLGGVPGGV.A |
| 7358 | 655 – 673 | 726.8616 | 1451.7087 | 1451.7158 | -4.88 | 0 | 20 | 0.27 | 1 | U | L.GGPGGFGGPGGLGGVPGGV.A |
| 1469 | 664 – 675 | 457.2404 | 912.4662 | 912.4665 | -0.41 | 0 | 26 | 0.46 | 1 | U | P.GGLGGVPGGVAG.G + Oxidation (P) |
| 1470 | 664 – 675 | 457.2410 | 912.4674 | 912.4665 | 0.99 | 0 | 27 | 0.63 | 1 | U | P.GGLGGVPGGVAG.G + Oxidation (P) |
| 2804 | 692 – 703 | 510.7593 | 1019.5041 | 1019.5036 | 0.44 | 0 | 20 | 1.2 | 2 | U | A.QYGLGGAGGLGA.G |
| 2807 | 692 – 703 | 510.7595 | 1019.5044 | 1019.5036 | 0.74 | 0 | 69 | 0.00013 | 1 | U | A.QYGLGGAGGLGA.G |
| 6953 | 692 – 708 | 688.3527 | 1374.6909 | 1374.6892 | 1.24 | 0 | 51 | 0.015 | 1 | U | A.QYGLGGAGGLGAGGLGA.G |
| 6954 | 692 – 708 | 688.3538 | 1374.6931 | 1374.6892 | 2.83 | 0 | 60 | 0.0018 | 1 | U | A.QYGLGGAGGLGAGGLGA.G |
| 9371 | 692 – 713 | 865.9412 | 1729.8679 | 1729.8748 | -3.98 | 0 | 88 | 1.3e-06 | 1 | U | A.QYGLGGAGGLGAGGLGAGGLGA.G |
| 9372 | 692 – 713 | 865.9444 | 1729.8742 | 1729.8748 | -0.31 | 0 | 97 | 9.9e-07 | 1 | U | A.QYGLGGAGGLGAGGLGAGGLGA.G |
| 9373 | 692 – 713 | 865.9457 | 1729.8769 | 1729.8748 | 1.25 | 0 | 73 | 2.1e-05 | 1 | U | A.QYGLGGAGGLGAGGLGAGGLGA.G |
| 9374 | 692 – 713 | 865.9460 | 1729.8774 | 1729.8748 | 1.53 | 0 | 50 | 0.0053 | 1 | U | A.QYGLGGAGGLGAGGLGAGGLGA.G |
| 10950 | 692 – 718 | 1043.5377 | 2085.0609 | 2085.0603 | 0.26 | 0 | 30 | 1.3 | 2 | U | A.QYGLGGAGGLGAGGLGAGGLGAGGLGA.G |
| 10951 | 692 – 718 | 1043.5381 | 2085.0616 | 2085.0603 | 0.61 | 0 | 27 | 0.03 | 1 | U | A.QYGLGGAGGLGAGGLGAGGLGAGGLGA.G |
| 11445 | 692 – 723 | 1221.1350 | 2440.2555 | 2440.2459 | 3.91 | 0 | 56 | 0.00021 | 1 | U | A.QYGLGGAGGLGAGGLGAGGLGAGGLGAGGLGA.G |
| 3509 | 694 – 708 | 542.7905 | 1083.5664 | 1083.5673 | -0.84 | 0 | 76 | 7.4e-05 | 1 | U | Y.GLGGAGGLGAGGLGA.G |
| 2061 | 699 – 711 | 478.7607 | 955.5068 | 955.5087 | -1.99 | 0 | 31 | 0.78 | 1 | U | A.GGLGAGGLGAGGL.G |
| 3510 | 699 – 713 | 542.7912 | 1083.5678 | 1083.5673 | 0.51 | 0 | 51 | 0.026 | 1 | U | A.GGLGAGGLGAGGLGA.G |
| 7296 | 699 – 718 | 720.3822 | 1438.7499 | 1438.7529 | -2.10 | 0 | 84 | 1e-06 | 1 | U | A.GGLGAGGLGAGGLGAGGLGA.G |
| 9918 | 699 – 723 | 897.9750 | 1793.9355 | 1793.9384 | -1.63 | 0 | 30 | 0.057 | 1 | U | A.GGLGAGGLGAGGLGAGGLGAGGLGA.G |
| 11289 | 704 – 734 | 1138.1134 | 2274.2123 | 2274.2081 | 1.83 | 0 | 47 | 0.0079 | 1 | U | A.GGLGAGGLGAGGLGAGGLGAGGLGAGGVIPG.A |
| 10479 | 709 – 734 | 960.5215 | 1919.0285 | 1919.0225 | 3.13 | 0 | 27 | 0.087 | 1 | U | A.GGLGAGGLGAGGLGAGGLGAGGVIPG.A |
| 8132 | 714 – 734 | 782.9227 | 1563.8308 | 1563.8369 | -3.94 | 0 | 42 | 0.0049 | 1 | U | A.GGLGAGGLGAGGLGAGGVIPG.A |
| 8134 | 714 – 734 | 782.9243 | 1563.8340 | 1563.8369 | -1.91 | 0 | 70 | 3.7e-05 | 1 | U | A.GGLGAGGLGAGGLGAGGVIPG.A |
| 956 | 735 – 744 | 414.2349 | 826.4552 | 826.4549 | 0.36 | 0 | 43 | 0.065 | 1 | U | G.AVGLGGVSPA.A |
| 957 | 735 – 744 | 414.2352 | 826.4558 | 826.4549 | 1.09 | 0 | 44 | 0.027 | 1 | U | G.AVGLGGVSPA.A |
| 8810 | 755 – 773 | 812.4511 | 1622.8875 | 1622.8893 | -1.08 | 0 | 27 | 0.074 | 1 | U | A.AGLGGVLGARPFPGGGVAA.R |
| 4936 | 756 – 768 | 599.3426 | 1196.6706 | 1196.6666 | 3.36 | 0 | 66 | 0.00022 | 1 | U | A.GLGGVLGARPFPG.G |
| 7168 | 756 – 771 | 705.8929 | 1409.7713 | 1409.7780 | -4.70 | 0 | 41 | 0.0045 | 1 | U | A.GLGGVLGARPFPGGGV.A |
| 7169 | 756 – 771 | 705.8930 | 1409.7715 | 1409.7780 | -4.62 | 0 | 47 | 0.00057 | 1 | U | A.GLGGVLGARPFPGGGV.A |
| 7170 | 756 – 771 | 705.8931 | 1409.7717 | 1409.7780 | -4.45 | 0 | 57 | 8.9e-05 | 1 | U | A.GLGGVLGARPFPGGGV.A |
| 7171 | 756 – 771 | 705.8933 | 1409.7721 | 1409.7780 | -4.18 | 0 | 55 | 0.00036 | 1 | U | A.GLGGVLGARPFPGGGV.A |
| 7597 | 756 – 772 | 741.4111 | 1480.8077 | 1480.8151 | -4.97 | 0 | 50 | 0.00053 | 1 | U | A.GLGGVLGARPFPGGGVA.A |
| 7598 | 756 – 772 | 741.4118 | 1480.8091 | 1480.8151 | -4.07 | 0 | 59 | 8.4e-05 | 1 | U | A.GLGGVLGARPFPGGGVA.A |
| 7599 | 756 – 772 | 741.4127 | 1480.8109 | 1480.8151 | -2.83 | 0 | 36 | 0.012 | 1 | U | A.GLGGVLGARPFPGGGVA.A |
| 7600 | 756 – 772 | 741.4131 | 1480.8116 | 1480.8151 | -2.34 | 0 | 21 | 0.15 | 1 | U | A.GLGGVLGARPFPGGGVA.A |
| 8023 | 756 – 773 | 776.9296 | 1551.8446 | 1551.8522 | -4.90 | 0 | 48 | 0.0059 | 1 | U | A.GLGGVLGARPFPGGGVAA.R |
| 8025 | 756 – 773 | 776.9299 | 1551.8452 | 1551.8522 | -4.51 | 0 | 50 | 0.0014 | 1 | U | A.GLGGVLGARPFPGGGVAA.R |
| 8027 | 756 – 773 | 776.9300 | 1551.8454 | 1551.8522 | -4.35 | 0 | 55 | 0.00075 | 1 | U | A.GLGGVLGARPFPGGGVAA.R |
| 8029 | 756 – 773 | 776.9313 | 1551.8480 | 1551.8522 | -2.70 | 0 | 43 | 0.0031 | 1 | U | A.GLGGVLGARPFPGGGVAA.R |
| 2894 | 758 – 768 | 514.2888 | 1026.5630 | 1026.5611 | 1.83 | 0 | 31 | 0.028 | 1 | U | L.GGVLGARPFPG.G |
| 5605 | 758 – 771 | 620.8426 | 1239.6706 | 1239.6724 | -1.46 | 0 | 32 | 0.081 | 1 | U | L.GGVLGARPFPGGGV.A |
| 5609 | 758 – 771 | 620.8433 | 1239.6720 | 1239.6724 | -0.38 | 0 | 45 | 0.0022 | 1 | U | L.GGVLGARPFPGGGV.A |
| 6545 | 758 – 772 | 656.3605 | 1310.7064 | 1310.7095 | -2.41 | 0 | 26 | 0.14 | 1 | U | L.GGVLGARPFPGGGVA.A |
| 6546 | 758 – 772 | 656.3643 | 1310.7140 | 1310.7095 | 3.37 | 0 | 30 | 0.049 | 1 | U | L.GGVLGARPFPGGGVA.A |
| 7019 | 758 – 773 | 691.8801 | 1381.7456 | 1381.7467 | -0.78 | 0 | 26 | 0.044 | 1 | U | L.GGVLGARPFPGGGVAA.R |
| 7020 | 758 – 773 | 691.8815 | 1381.7485 | 1381.7467 | 1.34 | 0 | 21 | 0.22 | 1 | U | L.GGVLGARPFPGGGVAA.R |
| 649 | 764 – 771 | 393.7151 | 785.4157 | 785.4184 | -3.51 | 0 | 25 | 0.088 | 1 | U | A.RPFPGGGV.A |
| 651 | 764 – 771 | 393.7161 | 785.4176 | 785.4184 | -1.10 | 0 | 22 | 0.2 | 1 | U | A.RPFPGGGV.A |
| 652 | 764 – 771 | 393.7161 | 785.4176 | 785.4184 | -1.10 | 0 | 25 | 2.7 | 3 | U | A.RPFPGGGV.A |
| 653 | 764 – 771 | 393.7162 | 785.4178 | 785.4184 | -0.79 | 0 | 28 | 0.13 | 1 | U | A.RPFPGGGV.A |
| 654 | 764 – 771 | 393.7164 | 785.4182 | 785.4184 | -0.25 | 0 | 34 | 0.064 | 1 | U | A.RPFPGGGV.A |
| 655 | 764 – 771 | 393.7168 | 785.4190 | 785.4184 | 0.68 | 0 | 48 | 0.028 | 1 | U | A.RPFPGGGV.A |
| 656 | 764 – 771 | 393.7170 | 785.4195 | 785.4184 | 1.31 | 0 | 45 | 0.047 | 1 | U | A.RPFPGGGV.A |
| 1134 | 764 – 772 | 429.2349 | 856.4552 | 856.4556 | -0.37 | 0 | 23 | 0.28 | 1 | U | A.RPFPGGGVA.A |
| 3739 | 772 – 782 | 551.3069 | 1100.5992 | 1100.5978 | 1.25 | 0 | 23 | 0.57 | 1 | U | V.AARPGFGLSPI.Y + Oxidation (P) |
| 10112 | 772 – 791 | 907.9810 | 1813.9475 | 1813.9475 | -0.023 | 0 | 39 | 0.023 | 1 | U | V.AARPGFGLSPIYPGGGAGGL.G |
| 10113 | 772 – 791 | 907.9841 | 1813.9537 | 1813.9475 | 3.41 | 0 | 22 | 0.23 | 1 | U | V.AARPGFGLSPIYPGGGAGGL.G |
| 10692 | 772 – 793 | 986.0261 | 1970.0376 | 1970.0374 | 0.080 | 0 | 53 | 0.00047 | 1 | U | V.AARPGFGLSPIYPGGGAGGLGV.G |
| 2740 | 773 – 782 | 507.7909 | 1013.5673 | 1013.5658 | 1.47 | 0 | 20 | 1.2 | 5 | U | A.ARPGFGLSPI.Y |

| Query | Start – End | Observed | Mr(expt) | Mr(calc) | ppm | M | Score | Expect | Rank | U | Peptide |
|---|---|---|---|---|---|---|---|---|---|---|---|
| 9459 | 773 – 791 | 872.4613 | 1742.9081 | 1742.9104 | -1.35 | 0 | 37 | 0.0052 | 1 | U | A.ARPGFGLSPIYPGGGAGGL.G |
| 10413 | 773 – 793 | 950.5074 | 1899.0003 | 1899.0003 | 0.018 | 0 | 27 | 0.071 | 1 | U | A.ARPGFGLSPIYPGGGAGGLGV.G |
| 1866 | 774 – 782 | 472.2721 | 942.5296 | 942.5287 | 0.99 | 0 | 21 | 1.2 | 2 | U | A.RPGFGLSPI.Y |
| 9113 | 774 – 791 | 836.9426 | 1671.8706 | 1671.8733 | -1.63 | 0 | 21 | 0.7 | 1 | U | A.RPGFGLSPIYPGGGAGGL.G |
| 10156 | 774 – 793 | 914.9898 | 1827.9651 | 1827.9632 | 1.02 | 0 | 47 | 0.0044 | 1 | U | A.RPGFGLSPIYPGGGAGGLGV.G |
| 10157 | 774 – 793 | 914.9901 | 1827.9655 | 1827.9632 | 1.29 | 0 | 60 | 0.00027 | 1 | U | A.RPGFGLSPIYPGGGAGGLGV.G |

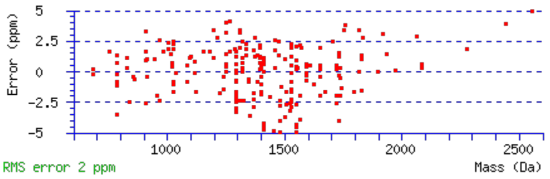


Mascot: http://www.matrixscience.com/
