# Supplementary figures and images for: A quantitative label-free analysis of the extracellular proteome of human supraspinatus tendon reveals damage to the pericellular and elastic fibre niches in torn and aged tissue
Source: PLoS One. 2017 May 18;12(5):e0177656. doi: 10.1371/journal.pone.0177656 (PMC5436668; doi:10.1371/journal.pone.0177656)

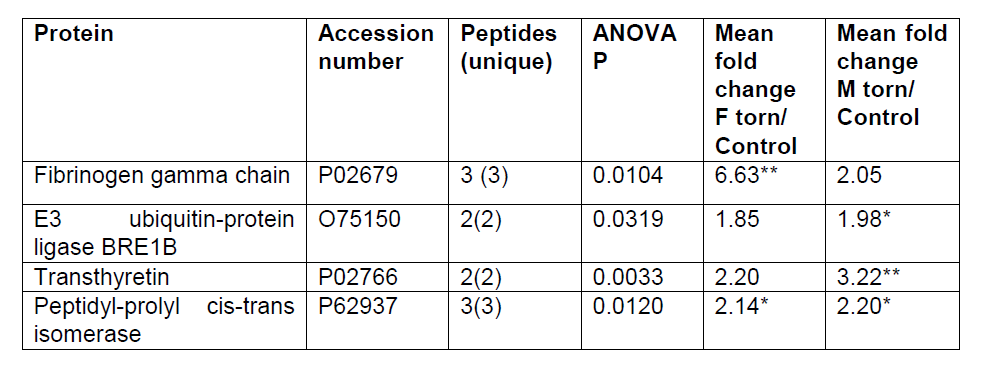

Supplement: S1 Table — (*P<0.05 **P<0.01 significantly different from control in post-test). (TIF) [file pone.0177656.s002.tif]

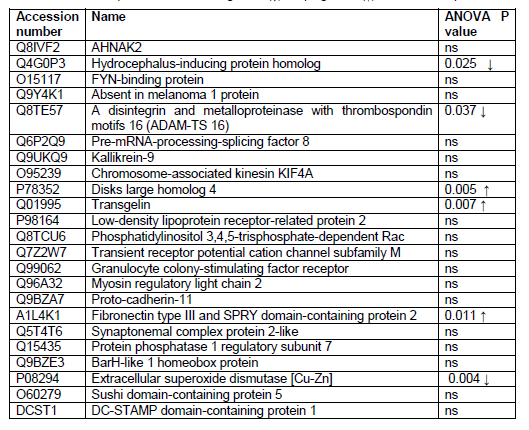

Supplement: S2 Table — (The arrows indicate if the proteins were downregulated (↓) or upregulated (↑) in the torn samples.). (TIF) [file pone.0177656.s003.tif]

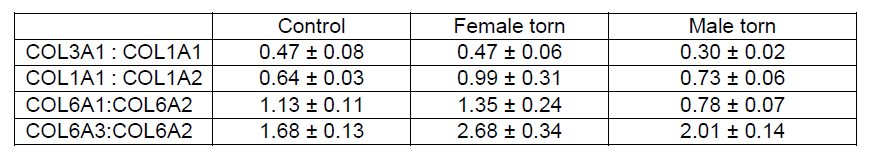

Supplement: S3 Table — (mean ± SEM, *P<0.05 significantly different from control). (TIF) [file pone.0177656.s004.tif]
